# Research progress on natural plant metabolites targeting apoptosis for endometriosis prevention and treatment: a systematic review

**DOI:** 10.3389/fphar.2025.1624569

**Published:** 2025-07-09

**Authors:** Yang Yu, Yufei Zhu, Mengyao Gao, Yu Wang, Shiying Li, Ying Gou

**Affiliations:** ^1^ Department of Obstetrics and Gynecology, The First Affiliated Hospital of Heilongjiang University of Traditional Chinese Medicine, Harbin, China; ^2^ Department of Obstetrics and Gynecology, Postdoctoral Mobile Station of Heilongjiang University of Traditional Chinese Medicine, Harbin, China; ^3^ Department of Obstetrics and Gynecology, Heilongjiang University of Chinese Medicine, Harbin, China; ^4^ Department of Obstetrics and Gynecology, Shaoxing Maternal and Child Health Hospital, Shaoxing, China

**Keywords:** natural plant metabolites, apoptosis, endometriosis, clinical translation, signaling pathways

## Abstract

Endometriosis (EMs) is a prevalent benign gynecological disorder characterized by dysmenorrhea and infertility, significantly impacting women’s health and quality of life. Currently, the pathogenesis of EMs remains incompletely elucidated. There are various side effects of drug treatment, while surgical interventions involve a certain degree of tissue trauma. Therefore, novel therapeutic drugs and clinical strategies should be developed. Apoptosis, a programmed cell death pathway, maintains tissue homeostasis, and its dysregulation is linked to various diseases and pathological complexities. Accumulating evidence suggests that abnormal apoptosis is intricately linked to the development and progression of EMs and that targeted promotion of cell apoptosis may contribute to the prevention and treatment of EMs. Natural metabolites, with their biological properties such as multipathway and multitarget effects, exhibit unique advantages in treating EMs, possibly by regulating apoptosis. This paper reviews the relationship between apoptosis and EMs. It systematically summarizes the latest progress of natural metabolites in treating EMS by regulating apoptosis, offering an innovative strategy for treating EMs.

## Graphical Abstract

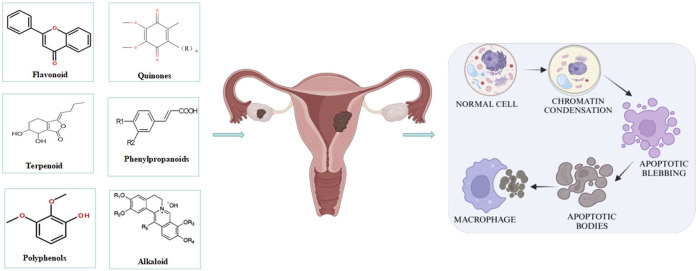

## 1 Introduction

Endometriosis (EMs) constitutes a prevalent chronic gynecological disorder characterized by the presence of functional endometrial glands and stroma outside the uterine cavity (Crump et al., 2024). Affecting 6%–10% of women of reproductive age (Zondervan et al., 2020), it typically presents with dysmenorrhea, chronic pelvic pain, and infertility. Epidemiological surveys have shown that the prevalence of EMs increases to approximately 30% among infertile patients and reaches up to 45% among those with chronic pelvic pain (Mounsey et al., 2006). In addition, although EMs is a benign disease, it exhibits typical features of malignant tumors, such as progressive and invasive growth, estrogen-dependent growth, and a tendency to recur and metastasize (Steinbuch et al., 2024). Various pathogenic mechanisms have been proposed for the development of EMs, including genetics, hormones, and immune factors. (Burney and Giudice, 2019; Saunders and Horne, 2021; Taylor et al., 2021; Vannuccini et al., 2022). In recent years, spontaneous apoptosis of the endometrium has been recognized as essential for maintaining its normal structure and function, whereas aberrant apoptosis promotes the development and progression of EMs (Yang et al., 2024).

Apoptosis, a distinct type of programmed cell death, is critical for eliminating damaged or unwanted cells (Zhong et al., 2024). Apoptosis is also pivotal in maintaining cellular homeostasis of tissues. Dysregulation of this process has been linked to diverse diseases and pathologies (Bertheloot et al., 2021), including neurodegenerative disorders, ischemic injuries, autoimmune diseases, and various types of cancer (Elmore, 2007). Under normal conditions, apoptosis destroys ectopic and eutopic endometrial cells before they form necrotic tissues, thereby preventing cell migration and accumulation (Agic et al., 2009; Taniguchi et al., 2011; Kobayashi et al., 2024). This underscores the critical role of apoptosis in the pathogenesis of EMs. Related studies have confirmed that in women with EMs, the eutopic endometrium shows increased expression of anti-apoptotic factors and decreased expression of pro-apoptotic factors, compared with the endometrium of healthy women (Harada et al., 2007). Such differences might facilitate the survival of refluxed endometrial cells within the abdominal cavity, thereby driving the progression of EMs. The potential of modulating apoptosis as a promising therapeutic approach for EMs has now been widely recognized. Therefore, an increasing number of studies are focusing on developing drugs to ameliorate EMs by promoting apoptosis.

In recent years, natural metabolites have emerged as an integral part of new drug development, especially those derived from plants. Natural metabolites treat EMs through a variety of molecular mechanisms, including pro-apoptotic, anti-inflammatory, anti-angiogenic, and antioxidant effects (Meresman et al., 2021). Systematic reviews and meta-analyses have further confirmed their efficacy, safety, and tolerability of natural metabolites, positioning them as a promising option for EMs. Numerous studies have indicated that natural metabolites are derived from a wide range of fruits and vegetables. Traditional Chinese medicinal plants are more suitable for long-term complementary and alternative therapies due to their low medication cost, fewer side effects, and higher safety profile (Xu et al., 2022). However, a large number of natural metabolites, such as quercetin, ginsenosides, curcumin, naringenin, and baicalein, show considerable potential to induce apoptosis. In light of this, this paper delves into the pathological mechanisms of apoptosis in EMs and explores natural metabolites targeting apoptosis for the treatment of EMs. Additionally, it discusses the limitations and challenges of this therapy. The aim is to provide novel strategies for developing therapeutic agents against EMs and meaningful references for related research.

## 2 Search strategy and selection criteria

We systematically searched PubMed, Embase, Web of Science, CNKI, Wanfang, VIP, and SinoMed databases using “endometriosis”, “apoptosis”, “natural compounds”, “natural products”, “Chinese herbal monomers”, “Chinese herbal extracts”, and related MeSH terms as keywords. Studies published between January 2004 and December 2024 were included, and the reference lists of eligible articles were further screened to identify additional relevant literature. Initially, 985 articles were identified through initial database searches. Subsequently, studies potentially affected by selection bias, detection bias, reporting bias, or other bias sources were excluded. Finally, 79 studies were considered eligible for inclusion in this review.

## 3 Major molecular mechanisms of apoptosis

**FIGURE 1:**
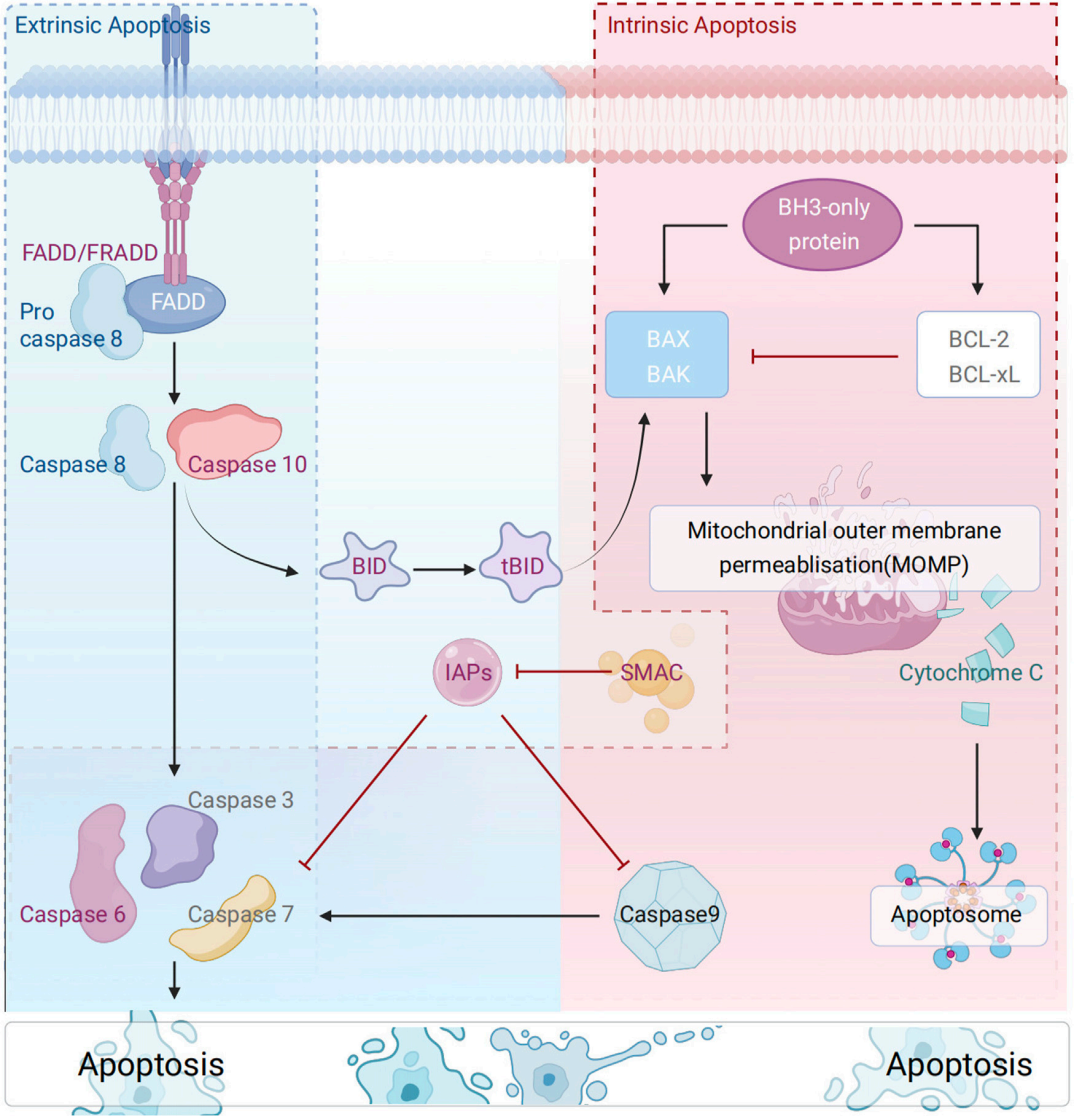
Schematic diagram of the molecular mechanism of apoptosis.

Apoptosis, a programmed death mechanism precisely regulated by genes, is central to the maintenance of organismal homeostasis (Mustafa et al., 2024). It is associated with a series of cellular morphological and functional changes, including cell shrinkage, chromatin condensation (accompanied by characteristic DNA fragmentation), plasma membrane blebbing, and the formation of phagocytotrophic apoptotic bodies (Bortner and Cidlowski, 2002; Dejas et al., 2023). Current studies have clearly defined two core apoptotic pathways: the mitochondria-dependent intrinsic pathway and the death receptor-mediated extrinsic pathway (Chinnaiyan, 1999; Dlamini et al., 2004; Yuan and Ofengeim, 2024). As confirmed by previous studies, the former is mediated by the dynamic homeostasis of the Bcl-2 family mediating the mitochondrial membrane permeability transition. Conversely, the latter activates the caspase cascade via the DISC complex; the two pathways ultimately converge at the co-activation of effector caspases (e.g., caspase-3/7) and the amplification of MOMP-dependent signaling, thus forming a sophisticated programmed cell death network (Figure 1).

The mitochondrial pathway is the central route of intrinsic apoptosis, whose progression is governed by the delicate balance between pro- and anti-apoptotic proteins of the Bcl-2 family (Kale et al., 2018). Functionally, Bcl-2 family proteins are classified into three subgroups: (1) anti-apoptotic proteins (e.g., Bcl-2, Bcl-XL, Bcl-W), (2) pro-apoptotic pore-forming proteins (e.g., Bax, Bak, Bok), (3) pro-apoptotic BH3-only proteins (e.g., Bad, Bid, Bik, Bim, Bmf, etc.) (Kim et al., 2009)., BH3-only proteins (key mediators of the mitochondrial pathway) regulate BAX/BAK activation through a dual mechanism: (1) direct activators (e.g., BID, BIM, PUMA) (Wei et al., 2000); (2) indirect regulators (e.g., BAD) (Wei et al., 2000; Czabotar et al., 2009; Huang K. et al., 2019).: Notably, recent studies have suggested that all BH3-only proteins can indirectly activate BAX/BAK by antagonizing BCL-xL/MCL-1 (Antignani and Youle, 2006), a hypothesis that warrants further validation and exploration. Furthermore, studies have shown that BAX/BAK activation and oligomerization during intrinsic apoptosis drive the formation of the mitochondrial outer membrane permeability transition pore (mPTP), triggering the release of mitochondrial contents (Ehrmann et al., 2023). This, in turn, activated and amplified apoptotic signaling. In the cytoplasm, SMAC (Second Mitochondrial Activator of Caspases)/OMI (also known as HtrA2) synergistically enhances apoptotic signaling by binding to X-linked inhibitor of apoptosis (XIAP) (Brentnall et al., 2013; Jost and Vucic, 2020). Additionally, stimuli such as DNA damage, oxidative stress, endoplasmic reticulum stress, and mitotic defects can activate this apoptotic cascade (Nuñez et al., 1990; Zou et al., 1997; Czabotar et al., 2014).

The extrinsic apoptotic pathway is initiated by the activation of death receptors (e.g., Fas/TNF-R1/DR4-5), which recruit adaptor proteins such as FADD (Fas-associated death domain protein)/TRADD (TNF receptor-associated death domain protein) (Nagata, 2018). Among them, FADD recruits procaspase-8 (cysteine precursor-8) to form a death-induced signaling complex (DISC) at the plasma membrane surface (Han et al., 2023). This facilitates the initiation of the apoptotic execution program. The phosphorylation status of RADD, a TNF-R1-specific adaptor protein, dictates the direction of apoptotic signaling. The execution of these processes is intricately linked to the TNF receptor superfamily, the core regulatory hub of extrinsic apoptosis. The TNF receptor superfamily specifically activates cysteine proteases of the caspase family via ligand-receptor binding, particularly the caspase-8 signaling axis (Chipuk et al., 2006; Hughes et al., 2016). In apoptosis, beyond the caspase-dependent pathway, mitochondrial outer membrane permeability (MOMP) leads to the release of apoptosis-inducing factor AIF (a mitochondrial oxidoreductase). AIF directly induces DNA fragmentation and chromatin condensation via the caspase-independent pathway, serving as a key node connecting the intrinsic and extrinsic apoptotic pathways. This factor directly induces DNA breaks and chromatin condensation through the caspase-independent pathway. Additionally, AIF serves as a key hub connecting the intrinsic and extrinsic apoptotic pathways (Lorenzo et al., 1999; Kuan et al., 2021).

Apoptosis can be subdivided into extrinsic and intrinsic types. Extrinsic pathway: The process commences with the binding of the death receptor to the receptor, resulting in the activation of the intracellular domain, or DD, of the death ligand. Through the activity of the adaptive molecules FADD and TRADD, a connection is established with the DED domain of the Caspase-8 proenzyme, resulting in the formation of the DISC complex. The proenzyme Caspase-8 undergoes self-catalysis to produce active Caspase-8. The stimulation of downstream effectors Caspase-3, -6, and -7 by Caspase-8 results in substrate breakage and triggers cellular death. Intrinsic pathway: Internal cellular injury causes apoptosis of Bcl-2 family members, including Bad, which enhances the porosity of the mitochondrial outer membrane and facilitates the release of cytochrome c (Cytc) from the intermembrane gap of the mitochondria. The released Cytc engages with Apaf-1 and Caspase9 proenzymes in the cytoplasm to create an apoptotic complex that autonomously catalyzes the activation of Caspase9. The activation of downstream effectors Caspase-3, -6, and -7 by Caspase-9 results in substrate breakage and triggers cellular death.

## 4 Apoptosis in the pathogenesis of EMs

Studies have shown that the endometrium achieves cyclic proliferation and shedding through tightly regulated cell proliferation and apoptosis mechanisms, which are crucial for maintaining normal physiological functions. Therefore, apoptosis is pivotal in maintaining the cyclic structural and functional stability of endometrial cells (Doroftei et al., 2021). However, aberrant apoptosis—characterized by decreased susceptibility of endometrial cells to apoptosis—enhances their ability to grow at ectopic sites. This may further promote the development and progression of EMs (Depalo et al., 2009). Consequently, the apoptotic mechanism is intricately tightly linked to EM pathogenesis, underscoring the importance of investigating the role of apoptosis in EMs.

Bcl-2 and Bax, key proteins of the mitochondrial pathway, are involved in regulating morphological and functional changes in the normal endometrium. The ratio of these two factors determines the kinetics of endometrial cell apoptosis (Johnson et al., 2005). Their aberrant expression leads to dysregulation of apoptosis in endometrial cells. The Bcl-2/Bax expression ratio in the ectopic endothelial tissues of patients is significantly elevated, interfering with mitochondrial cytochrome C release. This, in turn, blocks the activation of the caspase protease cascade, reducing the apoptosis rate and facilitating the survival, implantation, and infiltration of ectopic endometrium (Junqi and Yan, 2008; Chen et al., 2019). Concurrently, oxidative stress induced by excessive mitochondrial ROS is critical for apoptosis in EMs cells (Rossi et al., 2019; Yang et al., 2019; Assaf et al., 2022). PGC-1α and PGC-1β, which inhibit estrogen receptor β (ERβ) activation, promote Nrf2 expression. In EMs, sustained Nrf2 activation induces mitochondrial oxidative phosphorylation, leading to excessive oxidative stress that may affect apoptosis. As a result, apoptosis is also affected (Haider and Knöfler, 2009; Liao et al., 2019).

Pro-apoptotic receptors and their ligands on endometrial cell surfaces are also pivotal in the pathogenesis of EMs (Gogacz et al., 2017). Fas functions as a death receptor on activated cell surfaces, regulating NK cells and lymphocytes to induce apoptosis (Garcia-Velasco et al., 1999). Conversely, further studies have shown a stage-dependent decrease in Fas ligand (FasL) expression in the ectopic endometrium of EMs patients (Bellezza et al., 2018). The Fas-FasL axis may also enable ectopic endometrial cells to evade immune surveillance, thereby promoting disease progression. Furthermore, it has been confirmed that apoptosis in EMs cells is positively regulated by p53. In contrast, endometriotic cells exhibit a Warburg effect, which increased glycolysis suppresses apoptosis by inhibiting excessive ROS production (Leonte et al., 2023). In addition, under hypoxic conditions, aberrant autophagy in EMs impedes apoptosis. Collectively, multiple biological mechanisms have been implicated in EM-associated apoptosis and disease progression, including the classical mitochondrial pathway, mitochondrial oxidative stress, Fas receptor-mediated apoptosis, and hypoxic stress.

## 5 Role of natural metabolites in apoptosis in EMs

**FIGURE 2:**
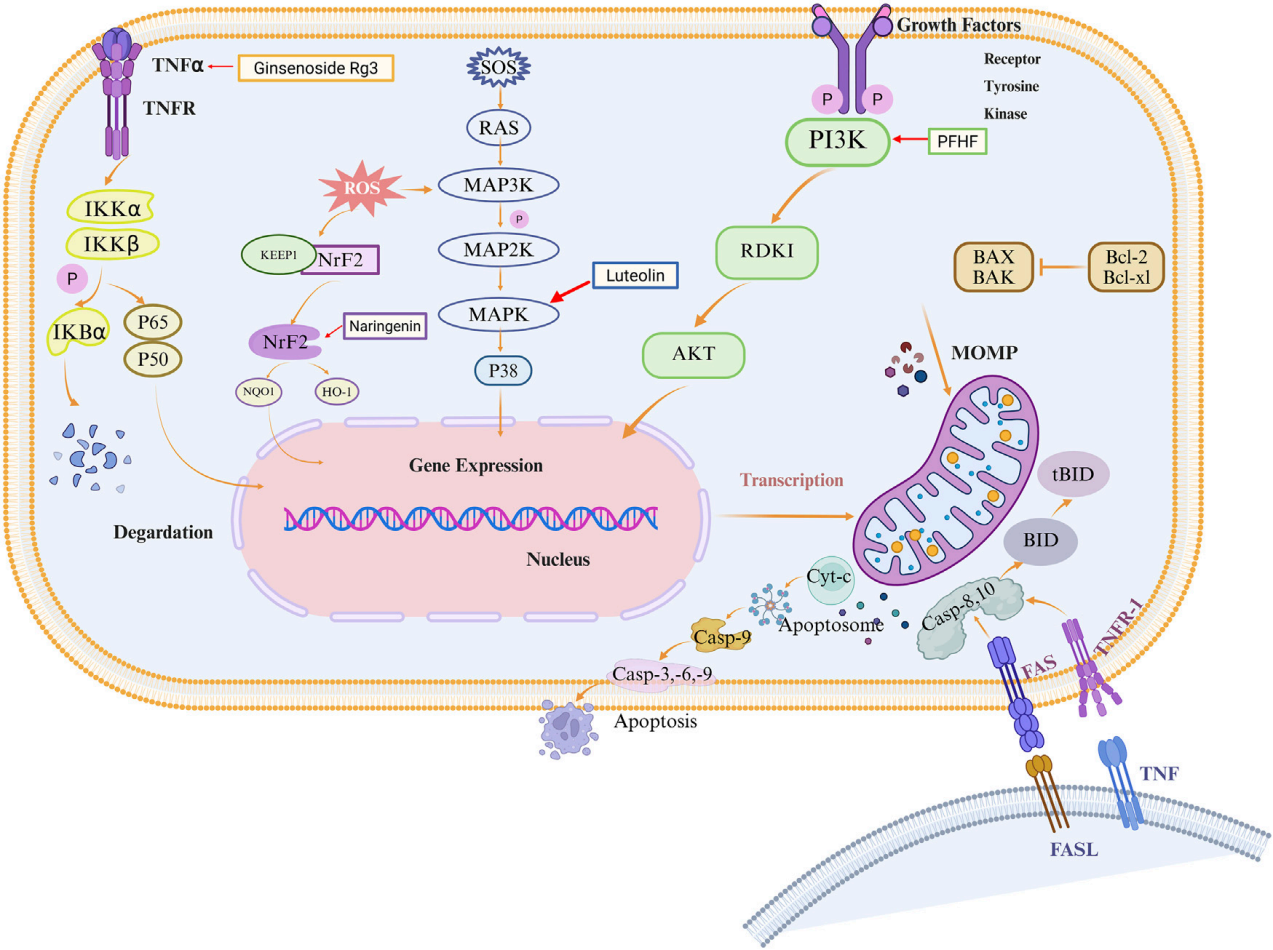
Schematic diagram of natural metabolites’ molecular mechanisms for the prevention and treatment of EMs by promoting apoptosis. Natural metabolites such as polyphenols, terpenes, quinones, phenylpropanoids, alkaloids, etc., mediate multiple signaling pathways such as MAPK, NF-κB, Nrf2, and PI3K/AKT, which induce intrinsic and extrinsic apoptosis in EMs cells.

Building on the established role of apoptosis in EMs pathogenesis, an expanding body of research has identified numerous natural metabolites with chemopreventive and therapeutic potential for EMs via apoptotic targeting. These apoptosis-promoting natural metabolites encompass a diverse array of plant-derived metabolites including terpenes, polyphenol quinones, phenylpropanoids, and alkaloids. Interestingly, by regulating MAPK, NF-κB, Nrf2, and PI3K/AKT signaling pathways as well as their unique molecular mechanisms to induce apoptosis, such natural metabolites exhibit multi-target and multi-pathway characteristics. Herein, we summarize naturally occurring apoptosis promoters and discuss their biological significance in EMs (Figure 2).

### 5.1 Polyphenols

Polyphenols, primarily derived from natural metabolites, are a group of phytochemicals containing hydroxyl groups and one or more aromatic rings, including flavonoids, tannins, phenolic acids, and anthocyanins (Goel et al., 2023). There is growing evidence that polyphenols from medicinal plants exert therapeutic effects through multiple biological activities such as free radical scavenging, antioxidant, anti-inflammatory, and pro-apoptotic activities (Wu et al., 2021; Li et al., 2022). Table 1 summarizes the mechanisms by which curcumin, resveratrol, quercetin, naringenin, baicalein, wogonin, and other polyphenolic metabolites improve endometriosis via apoptosis induction.

**TABLE 1:** Potential mechanism of polyphenols-induced apoptosis in the treatment of EMs.

Compound	In vitro*/In vivo*	Experimental model	Concentration	Pharmacological effects	Obstacles to development
Quercetin	In vitro*/In vivo*	VK2/E6E7 cells End1/E6E7 cells C57BL/6 mices	0, 5, 10, 20 μM 35 mg/kg	Inhibition of ERK1/P38/MAPK/AKT signaling pathway induces G0/G1 cycle arrested and promoted apoptosis in cells	Low water solubility and chemical instability
	In vitro	Endometrial stromal cells	25 µM	Mediated AKT-ERK-p53 signaling pathway increased apoptosis and aged-like phenotype of endometrial stromal cells	
	In vitro	End1/E6E7 cells	10, 20, 30, 40 μg/mol	Upregulation of miR-340-5p expression level significantly reduces cell OD value and PCNA, Bcl-2 protein levels, increased cell apoptosis rate, and protein expression of Bax and Caspases3	
	In vitro	End1/E6E7 cells	20 μg/mol	Upregulation of Bax expression and inhibition of Bcl-2 expression in combination with CADM1 induced an increase in Sub G0/G1 phase cells and a decrease in G0/G1 phase cells	
Naringenin	In vitro	VK2/E6E7 cells End1/E6E7 cells	0, 5, 10, 20, 50, 100 μM	Mitochondrial membrane depolarization and ROS metaboliteion induced cell apoptosis	Low bioavailability and significant first-pass effect
	In vitro	SD rats	0.5µM, 1.0µM, 5.0 µM	Inhibition of Nrf2/Keap1/HO1 signaling pathway induced mitochondrial membrane potential loss and ROS metaboliteion, leading to cell apoptosis	
Baicalein	In vitro	Endometrial stromal cells	0, 5, 10, 20, 40, 80, 160 µM	Activation of the NF-κB signaling pathway promoted cell cycle arrest in the G0/G1 phase and downregulated the expression of Bcl-2, PCNA, and cyclin D1 proteins	Low lipophilicity and hydrophilicity, significant first-pass effect, low intestinal absorption
	In vitro */In vivo*	Human ovarian endometriotic stromal cells	0–5 μg 40 mg/kg	Inhibited the MAPK/PI3K signaling pathway, increased mitochondrial calcium flux, induced mitochondrial depolarization and ROS, and promoted cell apoptosis	
Wogonin	In vitro*/In vivo*	Telomerase-immortalized Human Endometrial Stromal cells BALB/c mices	40, 80, 160 µM 20 mg/kg	Induced cell cycle arrest in the G2/M phase, increased intracellular ROS accumulation, and inhibited the expression of estrogen receptor alpha in cells	Low bioavailability and carrier stability check
	In vivo	SD rats	2 mg/kg, 14 mg/kg	Mediated the SIRT1/Nrf2 signaling pathway, upregulated the protein expression levels of SIRT1, Nrf2, GPX4, FTL, and SLC7A11,inhibited ferroptosis, and induced apoptosis	
EGCG	In vivo	SCID mice	50 mg/kg	Inhibited the expression of VEGF-A, HIF-1α, NF-κB, and MAP2K1 mRNA, reduced angiogenesis, and promoted cell apoptosis	Very low water solubility and fat solubility, insufficient oral bioavailability, and significant first-pass effect
	In vivo	BALB/c mices	20 mg/kg	Inhibited cell proliferation, reduced angiogenesis, and induced apoptosis	
	In vivo	Immunocompromised mices	50 mg/kg	Inhibited the growth of ectopic lesions and the functional and structural microvessels within the lesions, promoted apoptosis of the lesions	
	In vivo	C57BL/6 mices	50 mg/kg	Mediated Akt signaling pathway, inhibited angiogenesis, and induced apoptosis	
Puerarin	*In vito*	SD rats	80 mg/kg	Reduced E2 levels in ectopic endometrial tissue, upregulated ERβ expression, inhibited inflammatory processes, and promoted apoptosis	Low solubility, low permeability, very low bioavailability, very low bioavailability
	In vitro	Endometriotic stromal cells	1 × 10^−6^, 5 × 10^−6^, 1 × 10^−5^, 5 × 10^−5^, 1 × 10^−4^, 5 × 10^−4^ mol/L	Promoted the recruitment of estrogen receptors and restricted the recruitment of coactivators in ESCs, thereby downregulated the transcription of cyclin D1 and cdc25A, inhibited cell proliferation, and promoted cell apoptosis	
	In vitro	Endometrial stromal cells	100 μmol/L	Upregulated the gene expression of BAD, BAX, CASP8, CASP9, TNFRSF6, CDKN1B, CDKN2A, IFNA1, and IFNB1, downregulated the gene expression of FOS, CHEK2, SRC, ITGB5, MMP9, PDGFA, and NFKBIA, inhibited the formation of neovascularization in ectopic lesions, and promoted cell apoptosis	
Luteolin	In vitro	Human endometriotic 12Z cells	0, 15, 30, 60 µM	Stimulated the activation of Caspase-8, Caspase-9, and Caspase-3 in endometriosis cells and hindered the selective activation of macrophages	Low solubility, high permeability
	In vitro*/In vivo*	VK2/E6E7 cells End1/E6E7cellsC57BL/6 mices	0, 5, 10, 20, 50, 100 µM 40 mg/kg	Regulated the expression of PI3K/AKT and MAPK signaling proteins as well as CCNE1, blocked the cell cycle, inhibited cell proliferation, increased DNA fragmentation, and induced cell apoptosis	
Rutin	In vivo	Wistar albino rats	3,000, 6,000 μg/kg	Downregulation of Bcl-2, upregulation of Bax and caspase9 expression induced cell apoptosis, and improved oxidative stress by reduced MDA concentration and increased SOD, GPx, and TAC concentrations	Low intestinal absorption and poor oral bioavailability
	In vitro	CRL-7566 cells	70 μM	Targeted NOX4 and inhibited ROS/HIF-1 α signaling pathway, affected the malignant biological behavior of cells	
Silybin	In vitro*/In vivo*	VK2/E6E7 cells End1/E6E7cells C57BL/6 mice	0, 2.5, 5, 10, 25.50 µM 100 mg/kg	Promoted cell cycle arrest, oxidative stress, lipid peroxidation, and endoplasmic reticulum stress, thereby inducing cell apoptosis	Poor oral absorption
Chrysin	in vitro	VK2/E6E7 cells End1/E6E7 cells	0, 5, 10, 20, 50, 100 μM	Stimulated endoplasmic reticulum stress, ROS metaboliteion, and cytoplasmic calcium levels, downregulated PI3K signaling pathway transduction, and induced cell apoptosis	Low water solubility and first pass effect
Myricetin	In vitro*/In vivo*	VK2/E6E7 cells End1/E6E7 cells C57BL/6 mices	0, 5, 10, 20, 50, 100 μM 29 mg/kg	Downregulated the phosphorylation of ERK1/2 and PI3K/AKT signaling pathways, promoted G0/G1 phase arrest of cell cycle, inhibited cell proliferation, promoted mitochondrial dysfunction, accumulation of reactive oxygen species and calcium ions, and induced cell apoptosis	Low solubility and high rate of intestinal metabolism
Delphinidin	In vitro	VK2/E6E7 cells End1/E6E7 cells	0, 20, 50, 100 μM	Affected mitochondrial membrane potential and increased cytoplasmic calcium levels, thereby inducing cell apoptosis	Restricted intestinal absorption
Isoliquiritigenin	In vitro*/In vivo*	End1/E6E7 cells Balb/c mices	0, 25, 50, 75, 100 μM 1 mg/kg, 5 mg/kg	Inhibited the expression of Bcl-2 and increased the expression of Bax in endometriosis lesions, activated Caspase-3, promoted cell apoptosis, and inhibited the growth of endometriosis lesions	Poor targeting
Genistein	In vivo	SD rats	50, 150, 450 mg/kg/d	It may be related to the downregulation of Bcl-2, upregulation of Bax, and other related apoptotic factors, and at the same time, it inhibited other malignant activities, such as invasion and vascular proliferation of ectopic endometrium by inhibiting the expression of VEGF, CD34, COX-2, and MMP-9/TIMP-1	Low oral absorption
Curcumin	In vitro	Human ectopic endometrial cells	50 μmol/L	The percentage of cells in G1 phase increased and the percentage of cells in S phase decreased	Low water solubility, poor gastrointestinal stability, and rapid metabolism
	In vivo	Balb/c mices	12, 24, 48 mg/kg	Decreased Bax/Bcl-2 and induced the expression of Cyt-C and Caspase-9	
	In vivo	Balb/c mices	50 mg/mL	Downregulated of VEGF expression	
Resveratrol	In vitro	Human ectopic endometrial stromal cells	40–120 μM	Inhibits survivin mRNA expression and enhances trail, an apoptosis inducing ligand associated with TNF-α	Poor water solubility, poor photostability and strong first pass effect
	In vitro	Human ectopic endometrial stromal cells	100 μM	Increased Bcl-2/Bax gene expression	
	In vivo	SD rats	15, 45 mg/kg/d	Increased the expression level of Caspase-8, activated PPARα, and upregulated AMPK signaling and pcg1 pathway	
Paeonol	In vitro	Human ectopic endometrial stromal cells	0, 10, 30, 50, 100 μM	Downregulated LC3-II/LC3-I and Beclin-1, while upregulated p62	Poor water solubility, short peak time and fast drug absorption

#### 5.1.1 Flavonoids

**FIGURE 3:**
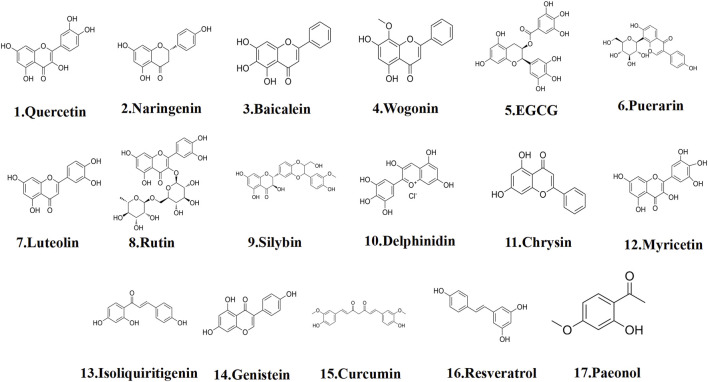
The structural formula of polyphenols.

Flavonoids are ubiquitously distributed in nature, predominantly existing in a glycosylated form in numerous medicinal plants, vegetables, and fruits (Bouyahya et al., 2021; Elham et al., 2021). Contemporary studies have confirmed their diverse pharmacological activities, including antioxidant, anticancer, anti-inflammatory, and cardiovascular protective effects (Fischer et al., 1997; Huang T. et al., 2019; Zhu et al., 2021). Regarding the treatment of EMs, several flavonoids, such as quercetin, naringenin, baicalein, and wogonin, exert therapeutic effects by inducing apoptosis, primarily through cell cycle arrest induction, endoplasmic reticulum stress activation, ferroptosis inhibition, and mitochondrial function disruption, thereby triggering apoptosis (Figure 3).

##### 5.1.1.1 Quercetin

Quercetin, a major dietary flavonol, is isolated from *Sophora japonica* L. (*syn. Sophora japonica* L.), is widely distributed in various common fruits and vegetables, such as *Allium cepa L.* (onion) and *Brassica oleracea* var. *botrytis* (cauliflower) (Ranganathan et al., 2015). As reported, it features various apoptotic effects in obesity, diabetes mellitus, cardiovascular disease, and a variety of tumor cells (Bishayee et al., 2013; Maciejczyk and Surowiak, 2013). Unsurprisingly, quercetin shows a significant pro-apoptotic potential to act as an apoptosis inducer in EMs. For example, Park et al. (Qingjing et al., 2023) observed *in vitro* experiments that 20 μM quercetin maximally blocked the cell cycle in a dose-dependent manner. This further resulted in a loss of mitochondrial membrane potential, DNA breaks, and reactive oxygen species production. Concurrently, an *in vivo* study revealed that CCND1 mRNA expression was substantially reduced after intraperitoneal injection (35 mg/kg) of quercetin. This stalled the cell cycle in the G0/G1 period and increased End1/E6E7 apoptosis, as compared to the blank group (Park et al., 2019a). Further studies have found that quercetin at a concentration of 25 μM could significantly enhance apoptosis and senescence-like phenotypes. It promoted the dedifferentiation of endometrial stromal cells in both control and endometriotic EMs. The underlying mechanisms involve two aspects: one is attenuating the phosphorylation of multiple signaling molecules in the AKT and ERK1/2 pathways, and the other is increasing the phosphorylation levels of p53 and the total amount of p53 (Delenko et al., 2024). In addition, quercetin might inhibit the proliferation of End1/E6E7 cells and promote cell apoptosis by upregulating miR-340-5p (Papari et al., 2020). Another *in vitro* study utilizing the identical cell model as the research object demonstrated that quercetin (20 μg/mol) promoted the expression of Bax and inhibited the expression of Bcl-2, This led to an increase in Sub G0/G1 phase cells and a decrease in G0/G1 phase cells, thereby inhibiting cell proliferation and inducing apoptosis (Jin et al., 2020). Nonetheless, certain investigations have indicated that the limited aqueous solubility and chemical instability of quercetin considerably restrict its absorption efficacy (Alizadeh and Ebrahimzadeh, 2022). Consequently, we can clarify the mechanism of pleiotropy by concentrating on the optimization of delivery systems and the resolution of toxicity mechanisms, while also integrating multi-omics techniques (e.g., transcriptomics and metabolomics) to elucidate pleiotropy and enhance the clinical application of quercetin-based versatile collaboration systems. In conclusion, quercetin may provide innovative ideas and targets as a treatment for EMs. Most current studies focus on *in vitro* and *in vivo* studies. However, the lack of clinical trials in humans precludes the elucidation of quercetin’s *in vivo* mechanisms.

##### 5.1.1.2 Naringenin

Naringenin (from *Ruta graveolens* L.) is predominantly found in *Citrus paradisi* Macfadyen (grapefruit) and *Citrus sinensis* (L.) Osbeck (sweet orange), and it constitutes a major phytochemical metabolite (Kawaii et al., 1999). It is a phytoestrogen featuring multiple effects, such as blocking the cell cycle, inducing ROS production, and inducing apoptosis (Lim et al., 2017). With research deepening, researchers focused on EMs and found that in the VK2/E6E7 and End1/E6E7 cell lines, naringenin targeted the MAPK and PI3K pathways in a dose-dependent manner. It upregulated pro-apoptotic proteins Bax and Bak via mitochondrial membrane potential depolarization, an increase in ROS production, and endoplasmic reticulum stress. As a result, it inhibited cell proliferation and promoted apoptosis (Park et al., 2017). In addition, naringenin significantly inhibited the expression of Nrf2 and its downstream effector molecules (NQO1, HO-1, etc.), causing a dose-dependent loss of mitochondrial membrane potential and inducing apoptosis (Kapoor et al., 2019). Naringenin, similar to quercetin, is characterized by low bioavailability and a pronounced first-pass effect (Cai et al., 2023). However, these problems may be solved by nanotechnology (e.g., liposomes), novel drug modifications, etc., in anticipation of its optimal clinical application (Vakilzadeh et al., 2021). Collectively, these results suggest naringenin’s pro-apoptotic potential in human EMs. However, evidence is mostly from *in vitro* studies, lacking *in vivo* data. It is anticipated to overcome current bottlenecks in molecular development and emerge as a promising natural drug candidate that have both therapeutic efficacy and safety.

##### 5.1.1.3 Baicalein

Baicalein (5,6,7-trihydroxyflavone), a flavonoid metabolite, is isolated from the roots of *Scutellaria baicalensis Georgi* (Lamiaceae) (Krawczyk et al., 2016). This metabolite exhibits multiple pharmacological properties multiple pharmacological properties, including anti-inflammatory, antibacterial, antiviral, antiallergic, antioxidant, and cytoprotective effects (Kim et al., 2005). A recent study revealed that treatment of human endometrial stromal cells with baicalein (40 µM) for 48 h resulted in a significant increase in the number of cells in the G0/G1 phase, as well as a significant decrease in the number of cells in the S phase and G2/M phase. Furthermore, the protein expression of Bcl-2, PCNA, and Cyclin D1 in the cells also decreased significantly. Activation of the NF-κB signaling pathway inhibits stromal cell viability, potentially inducing apoptosis (Jin et al., 2017). Existing studies have shown that iron accumulation can affect the expression of macrophage apoptosis genes and related proteins (HMOX1, FTH1, FTL) in ectopic tissues. Conversely, baicalein (20 µM) may increase the expression of GPX4, significantly inhibit ferroptosis, and then restore the phagocytosis of THP-1 cells, or induce apoptosis through this pathway (Yi et al., 2022). Due to its significant anti-inflammatory properties, baicalein reduced pro-inflammatory factor expression in ihOESCs. Additionally, it promoted apoptosis in these cells, increased mitochondrial calcium flux, and induced mitochondrial depolarization and ROS production (Park et al., 2024). Existing delivery systems can address its poor lipophilicity and hydrophilicity, significant first-pass effect, and lower intestinal absorption of baicalein due to the molecular structure of baicalein (Huang et al., 2014; Yu et al., 2017). Therefore, future studies should also integrate more precise delivery strategies to promote the efficient transformation of baicalein from natural metabolite to clinical drug.

##### 5.1.1.4 Wogonin

Wogonin (WG), derived from the same source as baicalein, has been shown to possess anti-tumor, anti-proliferative, anti-inflammatory, and pro-apoptotic properties in distinct studies (Li-Weber, 2009; Wu et al., 2016). WG constitutes one of the active ingredients in Wenjing Tang (a classic formula for treating EMs), which may treat endometriosis by modulating inflammation and endocrine secretion (Liu et al., 2021). *In vitro* studies confirmed that Treating THESC cells with WG (40 μM, 80 µM) for 24 h significantly inhibited cell proliferation, induced G2/M phase arrest in T-HESC cells, increased intracellular ROS accumulation, and inhibited the expression of estrogen receptor. Further, *in vivo* studies indicated that WG (20 mg/kg) notably reduced the size of ectopic lesions, decreased the proliferating cells, and increased the rate of apoptotic cells (Ferella et al., 2018). In addition, WG significantly ameliorated the histopathological damage of ectopic endometrial tissue and reduced the serum levels of E_2_, P, IL-1β, and IL-6 (Xiaoying et al., 2023). In contrast to the extensively studied baicalein, research on wogonin has primarily focused on conventional nanoparticulate formulations, with stability posing a more significant challenge (Yu et al., 2017; Yang et al., 2022). Collectively, both *in vivo* and *in vitro* experiments have convincingly demonstrated its therapeutic effects. Its increased clinical application is eagerly anticipated, which will help elucidate the safety and efficacy of this treatment.

##### 5.1.1.5 Epigallocatechin-3-gallate

Epigallocatechin-3-gallate (EGCG), found in *Camellia sinensis* (L.) Kuntze, demonstrates a variety of antioxidant, anti-angiogenic, and pro-apoptotic pharmacological effects (Zaveri, 2006; Khan and Mukhtar, 2008). Upon intraperitoneal injection of EGCG (50 mg/kg), it significantly downregulated the levels of VEGF-A and HIF-1α mRNA while up-regulating the levels of NF-κB and MAP2K1 mRNA, thereby promoting apoptosis in the foci and further reducing microvessel size and density resulting in a significant reduction of endometriotic foci, with a much smaller glandular epithelium, and an eccentrically distributed endometrium (Xu et al., 2009). In recent studies related to mouse models, this effect was also verified by EGCG (Ricci et al., 2013). While EGCG can effectively inhibit the occurrence and development of EMs, its bioavailability remains suboptimal. In response, the prodrug of EGCG (pro-EGCG, EGCG octaacetate) has been developed to improve the stability and bioavailability of EGCG *in vivo*. Findings from previous studies indicated that both EGCG and pro-EGCG significantly inhibited the growth of endometrial implants, disrupted the functional and structural microvasculature within the lesions, and promoted lesion apoptosis. Notably, pro-EGCG demonstrated a more potent effect (Wang et al., 2013). Using *in vitro* and *in vivo* gene knockdown assays and microvascular network imaging, recent research revealed distinct mechanisms of action between the two metabolites. While EGCG targeted the Akt pathway, pro-EGCG inhibited angiogenesis and exhibited stronger pro-apoptotic activities through the EGF/HIF-1α/VEGF pathway (Hung et al., 2024). These characteristics suggest that pro-EGCG may represent a novel anti-angiogenic therapeutic strategy for EMs. It holds promise for combination with existing clinical anti-angiogenic agents to enhance treatment efficacy.

##### 5.1.1.6 Puerarin

Puerarin, a phytoestrogenic isoflavonoid metabolite isolated from *Pueraria lobata* (Willd.) Ohwi, has demonstrated therapeutic potential in the management of cardiovascular diseases, alcohol use disorder, and neurodegenerative conditions (Atteritano et al., 2007). As observed, puerarin (80 mg/kg) not only reduced E2 levels in serum and ectopic endometrial tissue but also promoted E2 metabolism by up-regulating 17β-HSD2 expression and down-regulating 17β-HSD1 expression. It reduced ER binding in E_2_ endometrial cells, interfered with the binding of E2-ER complexes to transcriptional regulatory proteins as well as initiated gene transcription, and thus inhibited the growth of ectopic endometrial lesions (Dassen et al., 2007; Yu et al., 2015). Meanwhile, it revealed that puerarin inhibited the above proliferation, which might be achieved partially by promoting co-inhibitor recruitment of the estrogen receptor, as well as restricting coactivator recruitment in endometrial cells, and down-regulating the transcription of cyclin D1 and cdc25A (Ji et al., 2013). In addition, puerarin could upregulate Erβ expression, inhibit the inflammatory process, and promote its apoptosis (Yu et al., 2015). Moreover, another *in vivo* study showed that puerarin could inhibit the formation of neovascularization in ectopic lesions, reduce the blood supply to the ectopic lesions, and promote apoptosis by modulating gene expression of EMs-related oncogenes, cell cycle, and apoptosis factors, thus inhibiting the tumor-like malignant behavior of EMs endothelial cells (Chaoqin et al., 2009). Puerarin has been shown to have low solubility and low permeability, which has led to its extremely low oral bioavailability (Li et al., 2014). Future research should focus on structural modification, the development of penetration enhancers to promote its conversion from a natural metabolite to a clinical drug, and systematic toxicological evaluations in order to fully utilize its therapeutic potential for the treatment of EMs.

##### 5.1.1.7 Other flavonoids

In addition to the above flavonoid metabolites, several other natural metabolites associated with EMs apoptosis exist in nature, such as rutin, luteolin, total flavonoids of Polygala fallax Hemsl (PFHF), silibinin, delphinidin, chrysin, myricetin, isoglycoside (ISL), etc. All these have been shown to affect the apoptotic process in endometriosis in different ways. Rutin (from *S. japonica L.*) induced apoptosis by down-regulating Bcl-2 and up-regulating the expression of Bax and Caspase 9. It ameliorated oxidative stress and thus induced apoptosis by decreasing the concentration of MDA while elevating the concentration of SOD, GPx, and TAC (Talebi et al., 2021). In addition, rutin also inhibited endometriotic cell proliferation, migration, and invasion, and promoted apoptosis via a mechanism possibly related to targeting NOX4 and blocking ROS/HIF-1α signaling (Haoran et al., 2023). Similarly, luteolin (from *R. odorata* L.)inhibited the growth of 12Z human endometriotic cells and induced intrinsic apoptosis through activation of Caspase-3, Caspase-8, and Caspase-9 (Khazaei et al., 2016). Further *in vivo* studies confirmed that luteolin dose-dependently increased cell cycle arrest, enhanced DNA breaks, and induced apoptosis, thereby inhibiting the development of EMs (Park et al., 2019c). Moreover, C. Zhong et al. observed that 0.6 mg/mL PFHF significantly affected the malignant biological behaviors of human ectopic endometrial stromal cells after 24 h of treatment, which might induce apoptosis of EESCs through the PI3K/AKT signaling pathway (Zhong et al., 2023). Derived from *Silybum marianum* (L.) Gaertn, silymarin (from S. *marianum*), exerts pro-apoptotic, anti-proliferative, oxidative stress-inducing, and lipid peroxidation effects on human endometriosis cell lines by targeting the MAPK signaling pathway to induce endoplasmic reticulum stress (Ham et al., 2019). Similarly, chrysin induced programmed cell death through the aforementioned stress response (Ryu et al., 2019). Myricetin, a flavonoid metabolite derived from *Morella rubra* (Lour.), exhibits antiproliferative, antioxidant, and oxidative stress-inducing effects (Xie and Zheng, 2017). *In vivo,* studies of EMs have uncovered that it reduced lesion size in a mouse model of EMs by inhibiting Ccne1. *In vitro*, it induced apoptosis through inhibition of cell proliferation and cell cycle progression, as well as loss of mitochondrial membrane potential and accumulation of reactive oxygen species and calcium ions (Park et al., 2020). Concurrently, delphinidin (from *Vaccinium myrtillus* L.) exerted comparable effects on human endometrial cells. It modulated mitochondrial membrane potential and elevated cytoplasmic calcium levels in VK2/E6E7 and End1/E6E7 cells, ultimately triggering apoptosis (Park et al., 2019b). In contrast, another Isoliquiritigenin (ISL) isolated from *Glycyrrhiza glabra* L. could reduce the size of lesions, downregulate serum levels of inflammatory cytokines, inhibit the progression of epithelial-mesenchymal transition (EMT), and induce apoptosis (Gai et al., 2015; Hsu et al., 2020). In addition, Genistein (Gen), an isoflavone metabolite derived from *Glycine max* (L.) Merr., inhibits tumor cell proliferation and induces apoptosis (Hillman et al., 2007) This pro-apoptotic effect occurs in a dose-dependent manner, thereby slowing the progression of EMs. Interestingly, its mechanism of action might not involve the NF-κB signaling pathway. Instead, it could be associated with down-regulating Bcl-2, up-regulating the expression and activity of apoptotic factors such as Bax, and suppressing the invasion and angiogenesis of ectopic endometrium. This was achieved by inhibiting the expression of proteins such as VEGF, CD34, COX-2, and MMP-9/TIMP-1 (Huixing et al., 2014). Overall, most flavonoids are dependent on nano-delivery (e.g., PLGA, liposomes) or structural modifications (e.g., glycosylation, hydroxyethylation) due to poor solubility, first-pass effect, or limited intestinal metabolism (Li et al., 2023). Moreover, most of the available data are based on *in vitro* or short-term animal experiments, lacking long-term toxicology and metabolite safety assessment. We look forward to optimizing the perfect combination of the above substances with the existing formulation technology, which can be used in combination with existing drugs to expand the limits of their clinical applications (Liu F. et al., 2020). Undoubtedly, a variety of flavonoids can fully utilize their apoptosis-inducing ability in the treatment of EMs, which may provide more drug sources for the treatment of EMs and fully utilize their biological properties.

#### 5.1.2 Non-flavonoids

Curcumin is a natural polyphenolic metabolite extracted from *Curcuma longa* L. (Patel et al., 2020). Multiple studies have validated curcumin’s potential as an effective treatment modality for EMs. This highlights its ability to trigger programmed cell death in endometriotic tissues (Kamal et al., 2021). Cao et al. (Cao H. et al., 2017) showed that 50 μmol/L curcumin increased apoptosis rates in human endometriotic and endometrial stromal cells *in vitro* following 24 h treatment. Further studies revealed that intraperitoneal injection of curcumin (12, 24, and 48 mg/kg) decreased the expression of the anti-apoptotic factor Bcl-2 and downregulated the expression of Bcl-2 mRNA in endometrial cells of BALB/c mice (Yu and Shah, 2007; Liang et al., 2009). This could increase the Baw/Bcl-2 ratio, induce the expression of cytochrome c, Caspase 9, and p53, and accelerate apoptosis in EMs (Anto et al., 2002). Additionally, curcumin could abrogate EMs by inhibiting NF-κB translocation and MMP-3 expression (Jana et al., 2012). In contrast, resveratrol, a polyphenolic metabolite derived from *Reynoutria japonica* Houtt., exhibits potent apoptosis-inducing activity (Bhardwaj et al., 2007; van Ginkel et al., 2007). It was confirmed that resveratrol (50 mg/mL) caused a significant increase in apoptotic cells in ectopic lesions, while significantly reducing the volume and weight of endometriotic lesions (Taguchi et al., 2016). Conversely, resveratrol could cause apoptosis in endometriotic stromal cells by inhibiting survivin mRNA expression and enhancing TNF-α-related apoptosis-inducing ligand (TRAIL), an apoptosis-inducing ligand associated with TNF-α (Kolahdouz-Mohammadi et al., 2020). Resveratrol induced apoptosis by down-regulating Bcl-2 expression and decreasing the Bcl-2/Bax ratio in EuESCs and CESCs(Chen et al., 2021; Gołąbek-Grenda et al., 2023). Additionally, it promoted apoptosis by increasing the expression level of Caspase-8 through upregulation of signaling pathways such as AMPK and PCG1 (Zhang et al., 2019). Concurrently, the therapeutic potential of paeonol (from Paeonia lactiflor*a* Pall.) might be attributed to its antioxidant, antitumor, anti-inflammatory, and immunomodulatory properties (Erekat, 2018). A study showed that paeonol induced autophagy in ectopic endometrial stromal cells by down-regulating HIF-α. Hypoxia-induced autophagy was ameliorated by paeonol through down-regulating LC3 and Beclin-1 and up-regulating P62 (Pang et al., 2021), suggesting that paeonol inhibits autophagy-mediated cell death in the development of EMs. In addition, paeonol dose-dependently reduced the cell viability, proliferation, migration, and invasion of ectopic endometrial stromal cells. It also promoted apoptosis, thereby inhibiting the malignant biological behavior of the cells (Bolton and Dunlap, 2017). In conclusion, polyphenolic natural metabolites show promise as apoptosis inducers for EMs but face clinical translation barriers due to physicochemical properties (solubility/stability) and metabolic complexity. In the future, it is necessary to combine structural optimization and targeted delivery technologies, and strengthen the safety and dose-toxicity correlation studies of nanocarriers (Kong et al., 2024).

### 5.2 Terpenoids

**FIGURE 4:**
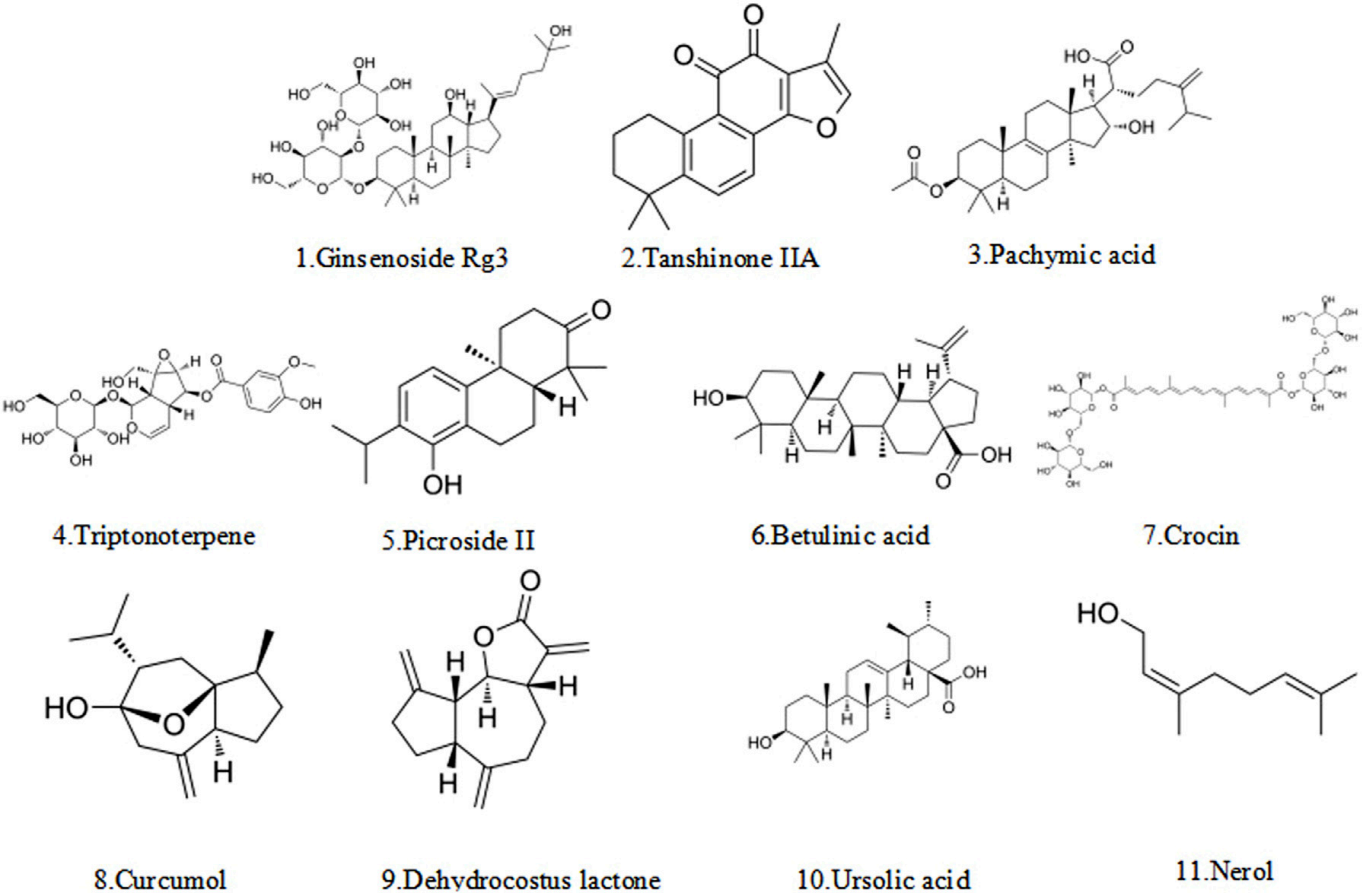
The structural formula of terpenoid.

Terpenoids, as a common class of metabolites in nature, are extensively found in a variety of medicinal plants (Baunach et al., 2015; Lu X. et al., 2023). Their specific typology is primarily shaped by the number of their carbon atoms in monoterpenes, diterpenes, triterpenes, etc. (Figure 4) (Huang et al., 2023). Terpenoids have demonstrated significant potential in the treatment of endometriosis by virtue of their ability to induce apoptosis in multiple ways. Table 2 summarizes their multiple core mechanisms, such as mitochondrial apoptosis pathway regulation and ferroptosis regulation, which trigger cell apoptosis. Moreover, representative metabolites like ginsenosides and pachymic acid can achieve therapeutic synergy through the above-mentioned multi-target synergistic regulatory network.

**TABLE 2:** Potential mechanism of terpenoids induced apoptosis in the treatment of EMs.

Compound	In vitro*/In vivo*	Experimental model	Concentration	Pharmacological effects	Obstacles to development
Ginsenoside Rg3	In vitro	Ectopic endometriotic stromal cells	25, 50, 100, 150 μg/mL	The mRNA expression of NF-κB P65,IL-8 and CIAP-2 was decreased to induce apoptosis	Poor water solubility,low bioavailability, easily degraded and difficult to optimize dosage
	In vitro	Endometrial cells	0.313 × 10^4^, 0.625 × 10^4^, 1.25 × 10^4^, 2.5 × 10^−4^, 3.75 × 10^−4^ mol/L	Induced cell G0/G1 cycle arrest and promoted cell apoptosis	
	In vitro	Ectopic endometriotic stromal cells	0, 25, 50, 100, 150 μg/mL	Upregulated the expression of Caspases3 and downregulated the expression of VEGF	
	In vivo	SD rats	5 mg/kg, 10 mg/kg	The PI3K/Akt/mTOR signaling pathway was blocked, and the expression of VEGF, p-Akt and p-mTOR was downregulated	
Tanshinone IIA	In vivo	SD rats	30 mg/kg	TGF-β/SMADS signaling pathway was inhibited, Vegf, Mmp9 and Bcl2 mRNA expressions were downregulated, and Bax and Caspase9 mRNA expressions were upregulated	Poor water solubility, low bioavailability and difficulty in dose optimization
	In vivo	SD rats	10, 20, 30 mg/kg	It inhibited the expression of Bcl-2 protein and promoted the expression of Bax and Caspase-9 protein	
	In vitro	Ectopic endometrial stromal cells	10, 20, 40, 80, 160 μM	Apoptosis was mediated by a dependent reduction of 14-3-3ζ	
Sodium tanshinone IIA sulfonate	In vivo	Balb/C mice	40–80 mg/day	The activation of p53, Sav1, and CCN1, coupled with the inhibition of HAS2, GM-CSF, and other cytokines, promoted cellular apoptosis	
Pachymic acid	In vivo	Sprague-Dawley rats	3.5 mg/kg, 7.0 mg/kg	Activated AMPK/GSK-3β/Nrf2 signaling pathway, reduced MDA, TNF-α and IL-6 levels, inhibited the expression of ACSL4 and PTGS2 proteins, and promoted the expression of GSH, p-AMPK/AMPK, p-GSK-3β/GSK-3β and Nrf2	Low levels in nature, low bioavailability, and toxicity and dose relationship not yet defined
Picroside Ⅱ	In vivo	SD rats	5 mg/kg, 10 mg/kg, 30 mg/kg	The expression of Bax, Bcl-2, VEGF and TGF-β was inhibited, and the number of CD206+ macrophages was decreased	Low bioavailability, toxicity and dose relationships have not been clarified
Triptonoterpene	In vitro	Endometrial stromal cells	20, 40 μg/mL	Increased KISS-1 mRNA expression, inhibited Ki67 and Pro-Caspase3 expression, promoted Cleaved Caspase3 and Bax protein expression	Poor water solubility, low bioavailability, and difficult dose optimization
Betulinic acid	In vitro	Human Endometriotic Epithelial Cell Line 12Z, Primary endometriotic epithelial cells	0, 5, 10, 15, 20, 25, 30, 35, 40 μM	Targeted genes associated with Estrogen Receptor β, including SOD2, NRF, COX2, and MMP1, effectively inhibited the metaboliteion of pro-inflammatory cytokines	Low solubility, low bioavailability, and difficult dose optimization
Neroli oil	*Bitter orange tree*	*In vivo*	40 mg/kg	Increased SOD, CAT levels, decreased NO, TNF-α, IL-8, IL-10, VEGF levels	Low water solubility, first pass metabolism, and potential toxicity of metabolites
Crocin	In vitro/In vivo	Human monocyte THP-1 cell Balb/c mice	20 μM 25 mg/kg	Reduced the release of VFP, IL-6, TNF-A and other cytokines, and induced cell apoptosis	Insufficient stability, low bioavailability
Curcumol	In vitro/In vivo	Ectopic endometrial stromal cells Sprague-Dawley rats	5–40 μg/L 20 mg/kg	Targeted JAK2/STAT3 pathway, inhibited the expression of Bax, caspase-3, TNF-α, IL6 and ILL-1L, and promoted the expression of Bcl2 protein	Low solubility, low bioavailability, and difficult dose optimization
Dehydrocostus lactone	In vitro	Human endometriotic cell line (12Z)	5, 10, 20 μM	Targeted the Akt and NF-κB pathways leads to the activation of caspase-3, caspase-8, and caspase-9, a reduction in the metaboliteion of BDNF, NGF, NT-3, and NT-4/5, and an inhibition of the expression of macrophage M2 markers, including IL-10, VEGF, MMP-2, and MMP-9	Low water solubility, low bioavailability, non-selective binding, and difficult dose optimization
Ursolic acid	In vitro	Ectopic endometrial stromal cells	15, 30, 45, 60 μM	Promoted the activity of caspase-3 and inhibited the expression of COX-2, PGE2 and VEGF	Poorly soluble and rapidly metabolized in the liver and gastrointestinal tract

#### 5.2.1 Ginsenoside Rg3

Ginsenoside Rg3, a terpenoid metabolite obtained from *Panax ginseng* C.A.Mey., exhibits multiple pharmacological effects including immunomodulation, fatigue alleviation, cardiomyocyte protection, and anti-diabetic and anti-cancer activities (Liu X. et al., 2020). It significantly inhibited endometrial stromal cells’ proliferation and induced apoptosis to inhibit EMs by inducing cell cycle blockade (Liqing et al., 2018). Similarly, low-to-medium concentrations (0.625 × 10^−4^ mol/L) enhance apoptosis, whereas medium-to-high concentrations (1.25 × 10^−4^ mol/L, 1.25 × 10^−4^ mol/L) induced G1-phase arrest and inhibit mitosis (Shaohui and Zhaoai, 2019). An in-depth study of the mechanism suggested that it might, on the one hand, act on the PI3K/Akt/mTOR signaling pathway to downregulate the expression of VEGF, p-Akt, and p-mTOR, inhibit angiogenesis, and promote cell apoptosis (Cao Y. et al., 2017). On the other hand, it might be closely related to the conventional NF-κB signaling pathway, decreasing the expression of NF-κB p65, IL-8, cIAP-2 mRNA, and VEGF, upregulating the expression of Caspases 3, and increasing the rate of apoptosis in endometrial stromal cells (Huang et al., 2020). In summary, ginsenoside Rg3 has the potential to serve as a precise natural lead compound for the treatment of EMs. The advancement of ginsenoside Rg3 as a medicinal pharmaceutical encounters numerous obstacles: The compound’s inadequate water solubility results in diminished bioavailability, evidenced by a total bioavailability of merely 2.63% in rat studies (Xie et al., 2005), making it difficult to reach an effective therapeutic concentration. In addition, its potential toxicity should not be ignored, and high doses may cause gastrointestinal discomfort and hepatotoxicity (Kim et al., 2018). Future development of innovative drug delivery methods is essential to enhance bioavailability and minimize toxicity, accompanied by comprehensive clinical trials to assess their effectiveness and safety.

#### 5.2.2 Tanshinone IIA

Tanshinone IIA (from *Salvia miltiorrhiza* Bunge) has multiple anti-inflammatory and antioxidant activities (Long et al., 2019). Intraperitoneal injection of Tanshinone IIA (30 mg/kg) significantly improved the symptoms of EMs, while reducing the volume of ectopic tissues in rats. This was achieved by inhibiting the TGF-β/SMADS signaling pathway, down-regulating the expression of cytokines, such as VEGF, MMP9, Bcl2, and TGF-β1, and up-regulating the expression of apoptotic proteins, such as Bax and Caspase 9 (Jianghong et al., 2016; Zhongling et al., 2021). In addition, it was also observed to decrease 14-3-3ζ, a key protein for maintaining cellular homeostasis, thereby promoting apoptosis (Wan et al., 2015). Sodium tanshinone IIA sulfonate (STS), a derivative of Tanshinone IIA, overcomes its water solubility limitation (Zhou et al., 2019). STS shranks lesions and activated p53, Sav1, and CCN1. Meanwhile, it inhibited factors such as HAS2 and GM-CSF, which in turn induced apoptosis (Luo et al., 2020). Despite its therapeutic potential, Tanshinone IIA faces clinical development challenges due to poor water solubility, leading to low oral bioavailability (Zhang et al., 2012). However, this challenge is expected to be addressed through novel formulations, such as solid lipid nanoparticles (SLN), which can significantly improve the bioavailability of tanshinone IIA.

#### 5.2.3 Triptonoterpene

Triptonoterpene is a major diterpenoid metabolite derived from *Tripterygium wilfordii* Hook. f. (Zhang et al., 2021). Data showed that the cell activity of Triptonoterpene (20 and 40 μg/mL) was significantly reduced upon treatment of the *in situ* mesenchymal endometrial cells for 48 h in a dose-dependent manner. Subsequent studies found that it inhibited cell proliferation and promoted apoptosis by increasing KISS-1 mRNA expression. This led to reduced Ki67 and Pro-Caspase3 levels while boosting Cleaved-Caspase3 and Bax protein expression (Xiao et al., 2002; Liling et al., 2021). Additionally, network pharmacological analysis of the main active substance in triptonoterpene revealed that it could act on several key signaling pathways of EMs. Meanwhile, it could also target the core of the treatment of EMs and give full play to its therapeutic effects (Dongfang et al., 2024). However, it's their physicochemical properties that lead to their limited absorption and distribution in the body. Meanwhile, the toxicity of triptonoterpene should not be ignored, as high doses may cause gastrointestinal discomfort, skin rash, central nervous system depression, and hematologic toxicity (Sun et al., 2013). Future research should focus on developing innovative drug delivery systems with precise dosage control to enhance their bioavailability and mitigate toxicity and conducting more extensive clinical trials to determine their safety and efficacy in humans.

#### 5.2.4 Other terpenoids

In nature, numerous terpenoid metabolites are closely associated with the apoptosis of EMs cells. Lin et al. (Lin et al., 2024) demonstrated that pachymic acid (from *Wolfiporia cocos* F.A.Wolf) could reduce the volume of ectopic endometrium, inhibit cell proliferation, attenuate inflammatory responses, and regulate ferroptosis. It also might decrease the levels of MDA, TNF-α, and IL-6, inhibit ACSL4 and PTGS2 protein expression, and contribute to the expression of GSH. Moreover, the effect of a high dose of pachymic acid (7.0 mg/kg) seemed to be more pronounced. Picroside II, an iridoid glycoside metabolite derived from *Picris hieracioides* L., reduced adhesions in SD rats, inhibited angiogenesis and fibrosis, attenuated M2 macrophage polarization, and promoted apoptosis in ectopic endometrium. This might be achieved by inhibiting Bax, Bcl-2, VEGF, and TGF-β expression, remarkably decreasing the number of CD206+ macrophages (Jun et al., 2020). Chen et al. (Chen et al., 2022) demonstrated that Betulinic Acid (BA) is isolated from *Betula pendula* Roth, targets genes associated with estrogen receptor β (ERβ), including superoxide dismutase 2 (SOD2), nuclear respiratory factor-1 (NRF1), cyclooxygenase-2 (COX-2), and matrix metalloproteinase-1 (MMP-1). It could inhibit pro-inflammatory cytokines, enhance oxidative stress, impair mitochondrial function, and promote the apoptosis of endometrial ectopic cells (Xiang et al., 2020). Meanwhile, as a natural antioxidant and anti-inflammatory agent, neroli oil had been found to significantly increase SOD and CAT levels after injection in model rats, thereby delaying the progression of EMs (Canday et al., 2024). Nerol (from *Citrus* L.) is the main natural metabolite of neroli oil, triggers mitochondrial dysfunction, and induces apoptosis by enhancing Ca^2+^ and oxidative stress (Tian et al., 2018). These findings suggest that neroli oil may serve as an apoptosis inducer for treating EMs. The terpene metabolite crocin extracted from *Crocus sativus* L. promoted apoptosis of ectopic endometrial cells. Meanwhile, it also inhibited the release of cellular inflammatory factors such as VFP, IL-6, TNF-α, etc., relieved symptoms, and slowed down the process (Liu et al., 2018; Guo et al., 2021). Curcumol (from *Curcuma longa* L.), a common terpenoid natural metabolite, was observed to induce apoptosis of endometrial stromal cells via a mechanism tightly linked to the JAK2/STAT3 pathway. As a result, it inhibits Bcl-2 protein expression, activates Bax and Caspase-3, and suppresses inflammatory factors such as TNF-α, IL-6, and IL-1β, thereby affecting the malignant biological behavior of the cells and reducing lesion size (Li et al., 2019; Wang et al., 2022). In contrast to curcumol, the action mechanism of dehydrocostus lactone (from *Dolomiaea souliei* var. cinerea) in treating EMs might be achieved through inhibition of the Akt and NF-κB signaling pathways. This increased the expression of Caspase-3, Caspase-8, and Caspase-9, and reduced the metabolism of pain-related neurotrophic factors such as BDNF, NGF, NT3, and NT4/5. Moreover, the expression levels of macrophage M2 markers as well as IL-10, VEGF, MMP-2, and MMP-9 were impeded, and apoptosis in 12Z human endometriotic cells was induced (Woo et al., 2019). In addition, ursolic acid (UA) is a member of terpenoids derived from *Osmanthus fragrans* Lour. (Song et al., 2012), increased Caspase-3 activity and inhibited the secretion of COX-2, PGE2, and VEGF in a dose-dependent manner, aiming to achieve the pharmacological activity of inhibiting angiogenesis and cell proliferation while promoting apoptosis (Li et al., 2020). However, most terpenoids face development barriers due to poor water solubility and rapid hepatic/gastrointestinal metabolism. Several approaches have been applied to address these issues, such as the preparation of liposomes, the development of terpenoid-loaded nanoparticles, and other innovative drug delivery systems (Cahova et al., 2017). In addition, further studies in animal models are needed to clarify subacute/chronic toxicity, molecular mechanisms, and structural modifications for developing novel therapies based on sesquiterpene lactones or derivatives.

### 5.3 Quinones

**FIGURE 5:**
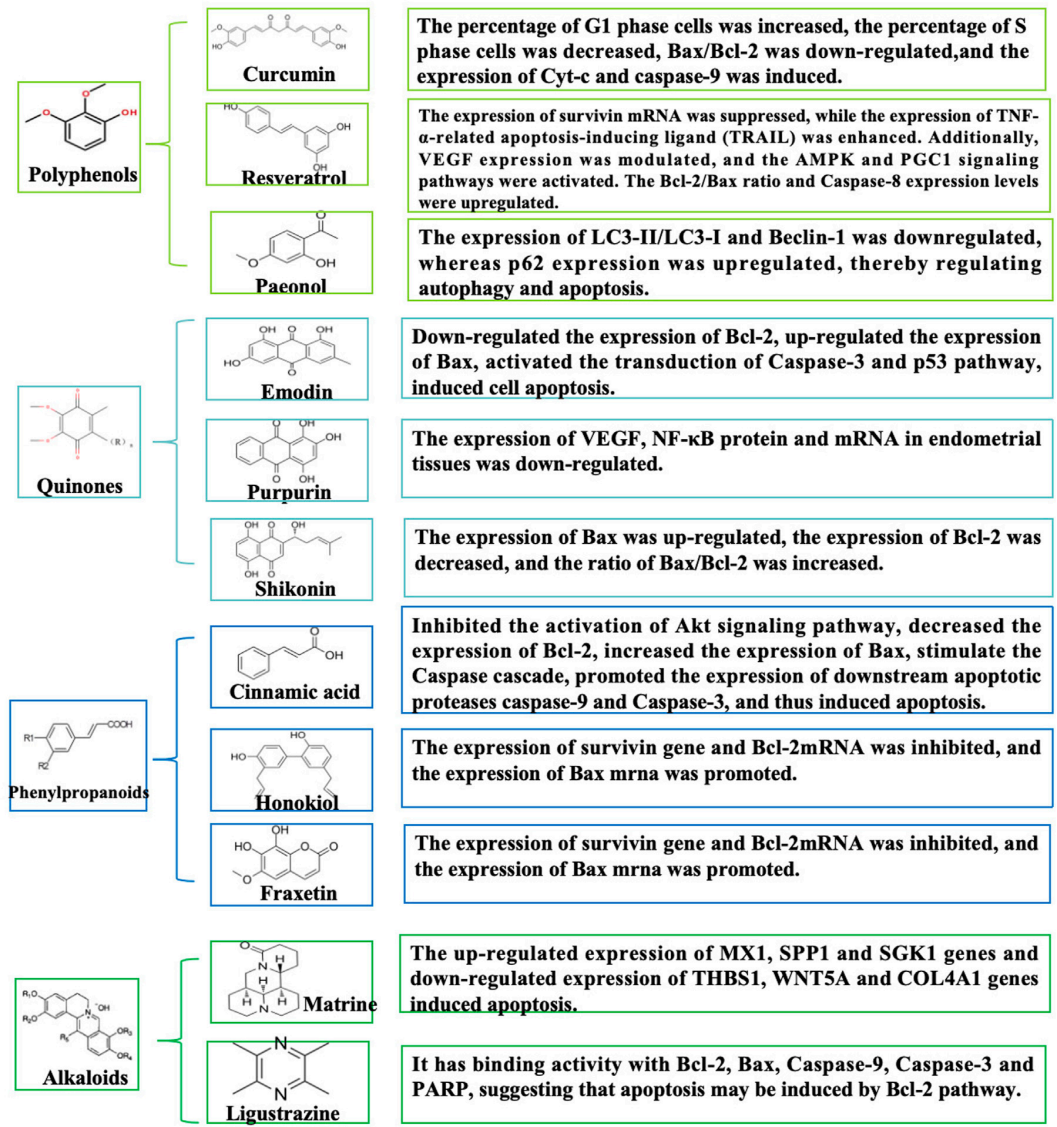
Schematic diagram of the mechanism of quinones, phenylpropanoids, and alkaloids in the treatment of EMs by promoting apoptosis.

Quinoid metabolites, naturally abundant in nature, comprise benzoquinones, naphthoquinones, anthraquinones, and phenanthrenequinones, with anthraquinones and their derivatives being particularly prevalent (Gomes de Carvalho et al., 2023). Emodin is a natural anthraquinone found in the *Rheum palmatum* L. and exhibits antimicrobial, anti-inflammatory, immunosuppressive, and anticancer effects (Zheng et al., 2020). Emodin induces apoptosis and inhibites invasion of ectopic endothelial and stromal cells via the downregulation of Bcl-2 expression, upregulation of Bax expression, and activating the Caspase-3 pathway (Zheng et al., 2016). Meanwhile, rhubarb-peach kernel herbal pair exerts anti-EMs effects by activating the p53 apoptotic signaling pathway, with emodin proposed as the key pro-apoptotic component (Liao et al., 2023). However, Zhang et al. (Yu and Hong, 2018) reported that shikonin, a naphthoquinone metabolite isolated from *Lithospermum erythrorhizon* Siebold and Zucc., enhances apoptosis in ectopic endometrial cells. This effect attenuated endometrial infiltration by modulating the Bcl-2/Bax ratio and accelerating ectopic cell apoptosis. The optimal efficacy was observed at a high shikonin concentration (5.6 mg/kg) (Hong and Yu, 2019). Collectively, these study results suggest that quinone-derived natural metabolites may fully activate the intrinsic pathway of apoptosis and show great potential for the treatment of EMs. However, quinone metabolites generally have clinical translation barriers such as poor water solubility, significant first-pass metabolism, and insufficient evidence of safety. Future research needs to focus on delivery system innovation (nano-delivery technology, colon-targeted delivery), optimization of structural modifications, establishment of humanized transgenic animal models, and simultaneous assessment of pharmacodynamic and metabolic regulatory effects and compound safety (Figure 5).

### 5.4 Phenylpropanoids

Phenylpropanoid metabolites have good biological activities in antioxidant, antitumor, endocrine, and other areas due to their structural uniqueness (Falcone Ferreyra et al., 2012). Among many phenylpropanoid metabolites, cinnamic acid, honokiol, and fraxetin can play a good role in EMs by inducing apoptosis. Cinnamic acid (from *Cinnamomum cassia* (L.) J. Presl) induces apoptosis in EESCs by downregulating Bcl-2 expression and upregulating Bax, thereby triggering the Caspase cascade reaction to promote downstream apoptotic proteases (Caspase-9 and Caspase-3). This process may also involve inhibition of the Akt signaling pathway (Hongli et al., 2021). Similarly, honokiol, a lignan metabolite obtained from *Magnolia officinalis* Rehder and E.H.Wilson, induces apoptosis by suppressing survivin and Bcl-2 mRNA levels while enhancing Bax mRNA expression (Wang et al., 2016). Furthermore, it was demonstrated that fraxetin (from *Fraxinus chinensis* Roxb.) disrupts calcium buffer homeostasis between mitochondria and ER and promotes intrinsic apoptosis during endometriosis (Ham et al., 2024). Most studies of phenylpropanoid analogs have focused on mitochondrial apoptosis, and the multiple interactions induced by phenylpropanoid metabolites have been under-analyzed, which may provide innovative paths for future studies. Most studies on phenylpropanoid compounds focus on mitochondrial apoptosis, and their clinical translation is limited by the triple dilemma of “absorption-metabolism-toxicity”, which requires breakthroughs in delivery technology innovation (e.g., smart nanocarriers), metabolic pathway regulation, and precise clinical trial design. Future research should prioritize establishing a standardized quality control system (e.g., USP certification) and developing a multi-omics biomarker network to facilitate their efficient translation from bench to clinic.

### 5.5 Alkaloids

Alkaloids, as a class of nitrogen-containing natural metabolites, exhibit diverse and remarkable biological activities (Qiu et al., 2014). As previously reported, alkaloidal natural metabolites such as matrine and ligustrazine have demonstrated potent apoptosis-inducing mechanisms, thereby inhibiting the progression of EMs. Specifically, the matrine metabolite from *Sophora flavescens* Ait., demonstrated a dose-dependent promotion of apoptosis in ectopic stromal cells, after which RNA-seq was used to identify differentially expressed genes. The most significantly differentially expressed upregulated genes included MX1, SPP1, and SGK1, and the downregulated genes included THBS1, WNT5A, and COL4A1 (Dandan, 2023). Ligustrazine, the main bioactive metabolite from *Ligusticum chuanxiong* Hort. (Apiaceae), exhibits significant efficacy in enhancing microcirculation, antithrombotic activity, sedation, and antioxidant defense (Qinglin et al., 2021). Further *in vitro* experiments showed that ligustrazine might inhibit the progression of EMs by down-regulating UBOX5-AS1 to inhibit EMs cells invasion, induce apoptosis, and mediate the activation of MMP-9/TIMP-3 signaling (Hongfang et al., 2023). Additionally, network pharmacological studies have revealed that ligustrazine, one of the main drug targets of Jiawei Foshou San (JFS), had binding activity with Bcl-2, Bax, Caspase-9, Caspase-3, and PARP in molecular docking experiments. This could possibly induce apoptosis through the Bcl-2 pathway and exert a positive effect (Wei et al., 2019). The primary obstacles to the clinical translation of alkaloids include dose-dependent organ toxicity (liver and kidney toxicity, neurotoxicity), low water solubility, first-pass metabolism, and multi-metabolite interference. Further integration of structural biology, nanotechnology, and computational toxicology is expected to break through the Activity-Toxicity-Delivery barrier of alkaloids and facilitate their transformation toward precision and intelligent drug development.

## 6 Clinical trials of natural metabolites for the treatment of EMs

The potential therapeutic efficacy of natural metabolites has been investigated in clinical trials, with some results have confirmed their effectiveness and safety (Table 3). However, these trials still exhibit significant limitations that compromise the reproducibility and generalizability of findings. First of all, the sample size of most clinical trials is small, which may cause the research results to be greatly affected by individual differences, limiting the statistical reliability of the research and making it difficult to obtain results with strong promotional value. Second, different trials have used varying administration methods (oral, local infiltration, sublingual, etc.) and dose regimens, making it hard to compare results between studies and affecting the stability of treatment effects. At the same time, the active ingredients of natural metabolites may be subject to extraction purity and stability, further affecting their controllability in the trial addition. The treatment cycles of trials varied widely, ranging from short-term (4 weeks) to long-term (1 year). This may lead to the long-term effect of clinical symptom improvement not being evaluated accurately, and it also makes the judgment of lesion progression more variable. Finally, although most trials use a Visual Analog Scale (VAS) to assess pain relief, the criteria for measuring lesion growth, hormone levels, quality of life, and other indicators have not been unified, making direct data comparison between different studies difficult. In order to further evaluate the value of natural metabolites in the treatment of EMs, future trials should consider several points to increase the sample size and improve the statistical power. The mode of administration and dose regimen were unified for cross-trial comparison. In addition, long-term observation indicators, including lesion progression, hormone level changes, and quality of life improvement, were set to comprehensively evaluate the clinical benefits of natural metabolites.

**TABLE 3:** Clinical trials of traditional natural metabolites for treating EMs registered on ClinicalTrials.gov.

Study title	NCT number	Status	Phase	Research progress
Evaluating the Impact of a Novel Cannabinoid metabolite for Endometriosis	NCT06477406	Recruiting	Phase II	The pain score (VAS), Beck Anxiety Inventory (BAI) and Beck Depression Inventory (BDI) of patients with cannabinoid endometriosis decreased significantly after use. The inflammatory factors IL-1, IL-6, IL-8, TNF-α were significantly decreased
Effect of Quercetin Supplementation on Endometriosis Outcomes	NCT05983224	Recruiting	Not Applicable	—
Cannabidiol for the Treatment of Pelvic Pain in Endometriosis (DREAMLAND)	NCT05670353	Recruiting	Phase III	The intensity of pain was significantly reduced, the change of pain threshold was significantly improved, and the scores of generalized anxiety disorder (GAD7) scale and depressive symptoms were decreased; There was no significant change in the concentration of alanine aminotransferase (ALT), aspartate aminotransferase (AST) and bilirubin in plasma
Cannabidiol and Management of Endometriosis Pain	NCT04527003	Terminated	Phase III	The pain intensity measured by cannabidiol visual analog scale was significantly improved
Green Tea Extract for Endometriosis Treatment	NCT02832271	Completed	Phase II	Green tea extract can reduce endometriosis lesions, reduce oral Rating Scale (ESS) and visual analogue scale (VAS) scores, and reduce the total number of new blood vessels in pathological tissues, without obvious side effects
Pertubation With Lignocaine in Endometriosis	NCT01329796	Completed	Phase II	Lignocaine can significantly reduce the visual analogue scale (VAS), however, the quality of life questionnaire remains to be evaluated

Note: As of 16 February 2025, a search for “condition/disease” on https://clinicaltrials.gov/using the keyword “Endometriosis” returned 756 studies. Among these, six clinical trials pertaining on natural metabolites were identified in the context of EMs treatment.

## 7 Discussion

EMs is a benign gynecological disorder characterized by the abnormal implantation of endometrial-like tissue outside the uterine cavity. The incidence of this disease has been rising steadily over the years. Statistics show that approximately 176 million women worldwide suffer from the disease (Swift et al., 2024). EMs can not only cause dysmenorrhoea, chronic pelvic pain, and infertility in women but also lead to mental health imbalances and affect the social functioning of the patient (Nnoaham et al., 2011). Considering EMs as an estrogen-dependent disease, the most common and effective pharmacological treatment is to inhibit the growth of intrinsic estrogen by altering gonadotropin-releasing hormone (GRH) (Giudice, 2010). However, these treatments are associated with significant side effects, are limited long-term applicability, and result in delayed conception (Lu J. et al., 2023). Therefore, new drugs and clinical strategies should be urgently developed to treat the disease based on innovative molecular mechanisms.

Apoptosis is a tightly regulated process of cell death, controlled by various stress modalities and complex molecular signaling pathways. Studies have indicated that aberrant apoptosis significantly contributes to the development and progression of EMs (Depalo et al., 2009) and that targeting apoptosis may provide novel targets, strategies, and pathways for the prevention and treatment of EMs. This paper presents a systematic and comprehensive review of the potential mechanisms of action of natural metabolites that ameliorate EMs by inducing apoptosis. It is confirmed that an increasing number of natural metabolites have been found to possess pro-apoptotic properties, which may hold promise for treating EMs. However, existing studies have certain limitations, particularly in the assessment of evidence quality and comparative analysis of results across different studies, which require further investigation. First, a variety of natural metabolites can induce apoptosis in EMs through overlapping signaling pathways. However, their differences in molecular regulation deserve further comparison. For example, quercetin and luteolin both modulate the MAPK signaling pathway, but quercetin induces apoptosis by inhibiting the ERK/P38/MAPK/AKT signaling axis, leading to DNA fragmentation, loss of mitochondrial membrane potential, and ROS accumulation. Luteolin, on the other hand, induced cell cycle arrest and promotes apoptosis mainly through the PI3K/AKT/MAPK signaling pathway. The primary distinction between the two lies in their specific molecular targets for inducing cell death, suggesting differential efficacy at distinct pathological stages of EMs. Meanwhile, naringenin, wogonin, and pachymic acid can modulate the Nrf2 pathway. Naringenin inhibites the Nrf2/Keap1/HO-1 signaling pathway, leading to enhanced oxidative stress, loss of mitochondrial membrane potential, and induction of apoptosis. Conversely, wogonin might inhibit ferroptosis and increase the percentage of apoptotic cells in EMs models by activating the SIRT1/Nrf2 signaling pathway. Pachymic acid mediated AMPK/GSK-3β/Nrf2 signaling pathway, reduces inflammatory response, regulates ferroptosis, and induces apoptosis of endometrial stromal cells. Although all of them can induce apoptosis, differences in their core molecular mechanisms may influence their clinical application strategies. It is worth noting that different compounds interact with the Nrf2 pathway via distinct mechanisms. It is expected that with further research, we can further clarify its mechanism of action. Meanwhile, metabolites such as curcumin and triptonoterpene, which are natural metabolites that mediate the NF-κB signaling pathway, have distinctive mechanisms in regulating apoptosis. Triptonoterpene induces apoptosis through the NF-κB and/or Rho-ROCK pathways, whereas curcumin not only activates the mitochondria-mediated apoptosis pathway but also downregulates the expression of Bcl-2 and upregulates Bax and Bad by inhibiting the NF-κB pathway. This mechanistic divergence suggests that curcumin may exert dual anti-inflammatory and pro-apoptotic effects, whereas triptonoterpene tends to induce apoptosis directly. Second, despite numerous studies highlighting the potential pro-apoptotic effects of natural compounds in EMs treatment, these findings must be interpreted with caution. Current research relies heavily on *in vitro* cellular models and *in vivo* animal experiments. However, *in vitro* experiments, constrained by artificially simulated environments and simplified cellular models, fail to adequately recapitulate the complex pathological milieu of the human body, particularly in domains such as drug metabolism, immune regulation, and endocrine interventions. Notably, certain natural metabolites like flavonoids (e.g., naringenin, silibinin, luteolin) and quinones (e.g., shikonin) have been categorized as pan-assay interference compounds (PAINS), metabolites susceptible to false-positive results in high-throughput screening, *in vitro* experiments. These PAINS compounds often often interact non-specifically with multiple targets, potentially leading to misleading “false activity” findings. For instance, flavonoids such as naringenin and silibinin are prone to non-specific interactions with diverse proteins due to the presence of functional groups such as phenolic hydroxyl moieties and conjugated double bonds in their structures, thereby compromising the physiological activation status of signaling pathways. Concurrently, Bolz et al. (Bolz et al., 2021) demonstrated that PAINS metabolites exhibit pronounced binding promiscuity and target non-selectivity in protein databases, which substantially exacerbates the misinterpretation of their “broad-spectrum activity” *in vitro* assays. Therefore, when interpreting the effects of such natural metabolites on EMs, it is critical to emphasize that “effective *in vitro*” does not equate to “clinically reliable”. In the absence of target validation and structural modifications, these metabolites are likely to be “chemical level disruptors” rather than genuine therapeutic candidates. Additionally, current studies are mainly based on *in vitro* cellular experiments and *in vivo* animal models. However, the translation of these experimental results to the clinic remains challenging. Information on the underlying molecular mechanisms is provided in the *in vitro* experiments, but the effects of drug metabolism, immune system effects, and endocrine modulation on the foci of EMs are neglected. For instance, quercetin exhibits significant pro-apoptotic effects in cell culture experiments, but its bioavailability may be reduced by metabolic processes *in vivo*. Although animal models can more comprehensively mimic the pathological process of EMs, physiological differences between species may lead to differences in drug efficacy. For example, curcumin demonstrates efficacy in mouse models, but its poor solubility and low bioavailability in human physiology limit its clinical utility. Meanwhile, the current number of clinical trials for natural compounds is limited, and there is a lack of systematic randomized controlled trials (RCTs) to verify their therapeutic efficacy and safety. Consequently, future studies should prioritize well-designed clinical trials to assess the true efficacy of these metabolites and to determine optimal therapeutic dosages.

To advance the clinical translation of natural compounds for EMs therapy, future research should focus on the following directions. First, during the screening of natural compounds, a PAINS screening filter should be integrated to identify and exclude potential interfering compounds using computational chemistry and structural biology approaches—before candidates enter animal or clinical trials. Meanwhile, although numerous studies have validated their efficacy, inconsistencies remain in evidence regarding specific mechanisms of action, key targets, and optimal dosing regimens. This suggests that we should pay more attention to the standardization of experimental conditions in subsequent studies, including the uniform selection of cell lines and standardization of dosage and cycle of drug administration. At the same time, strengthening the strategy of combined medication with different natural metabolites may improve the therapeutic effect through a synergistic effect. Secondly, drug delivery system optimization is critical. At present, the bioavailability of most natural metabolites is low. Nanotechnology (lipid nanocarrier, microemulsion technology, etc.) can be used to improve their stability, absorption rate, and pharmacokinetic characteristics, so as to improve their therapeutic effect. At this stage, the research on apoptosis of EMs induced by natural metabolites mainly involves *in vitro* cell experiments and *in vivo* animal experiments. Future efforts should prioritize multicenter RCTs to systematically evaluate the efficacy and safety of natural metabolites in EMs patients, accompanied by long-term follow-up to assess drug impacts on adverse reactions, recurrence rates, and quality of life. In summary, natural metabolites demonstrate promising pro-apoptotic potential in EMs therapy. However, current studies still lack in-depth comparative analysis and systematic evidence assessment, as well as systematic assessment of PAINS risk. Future studies should integrate critical PAINS mechanism-based evaluation, optimize drug delivery techniques, strengthen combination therapy strategies, and promote the development of clinical trials to enhance the practical application value of natural metabolites in the treatment of EMs.

## 8 Conclusion

This paper systematically summarizes the potential mechanisms by which diverse natural metabolites induce apoptosis in EMs therapy. Researches have shown that the effectiveness of these natural metabolites in treating EMs has been demonstrated *in vitro* and *in vivo* experiments. Various natural metabolites such as quercetin, luteolin, and naringin can regulate key signaling pathways (such as MAPK, Nrf2, NF-κB) to promote cell apoptosis, exhibiting multi-target and multi-pathway characteristics, providing new ideas and strategies for the treatment of EMs. However, current researches still have limitations, especially in terms of PAINS metabolites and clinical translation. Future researches should prioritize PAINS risk assessment, bioavailability enhancement, and advancement of multi-center randomized controlled trials to validate clinical efficacy and safety profiles.

## Glossary

AMPK: AMP-activated Protein Kinase
APAF-1: Apoptotic peptidase-activating factor 1
Bax: BCL2-Associated X protein
Bcl-2: B-cell lymphoma/leukemia-2
Caspases: Cysteine-requiring aspartate protease
CCNE1: Cyclin E1
DED: Death effector domain
DISC: Death-inducing signaling complex
EMs: Endometriosis
ER: Endoplasmic reticulum
ERK1/2: Extracellular signal-regulated kinase 1/2
FADD: Fas-associated death domain
FTH1: Ferritin Heavy Chain 1
FTL: Ferritin Light Chain
FOX: Forkhead box
GM-CSF: Granulocyte-macrophage colony-stimulating factor
GSK-3β: Glycogen synthase kinase 3β
GPX4: Glutathione Peroxidase 4
HNF-1β: Hepatocyte Nuclear Factor 1 beta
HO-1: Heme Oxygenase-1
IhOESCs: Immortalized human ovarian endometriotic stromal cell lines
LC3: Microtubule-associated Protein Light Chain 3
MAPK: Mitogen-activated protein kinase
MDA: Malondialdehyde
MLKL: Mixed lineage kinase domain-like pseudokinase
MMP-9: Matrix metalloproteinase-9
mTOR: mammalian target of rapamycin
NF-κB: Nuclear Factor kappa-B
NOX4: Nicotinamide Adenine Dinucleotide Phosphate Oxidase 4
NRF2: Nuclear factor erythroid 2-related factor 2
NQO1: Quinone Oxidoreductase 1
PAINS: Pan-assay interference compoundss
PCNA: Proliferating Cell Nuclear Antigen
PGC-1α: Peroxisome proliferator-activated receptor-γ coactivator-1α
PI3K/AKT: Phosphoinositide 3-kinase/Protein Kinase B
RIPK: Receptor-interacting protein kinase
ROS: Reactive oxygen species
SMAC: Second Mitochondrial Activator of Caspases
TGF-β1: Transforming Growth Factor Beta Receptor I
