# Supplementary material for: Research progress on natural plant metabolites targeting apoptosis for endometriosis prevention and treatment: a systematic review
Source: Front Pharmacol. 2025 Jul 9;16:1624569. doi: 10.3389/fphar.2025.1624569 (PMC12283756; doi:10.3389/fphar.2025.1624569)
Supplement: Supplementary file 1 [file DataSheet1.pdf]

Supplementary Material

1 Supplementary Figures and Tables

1.1 Supplementary Figures

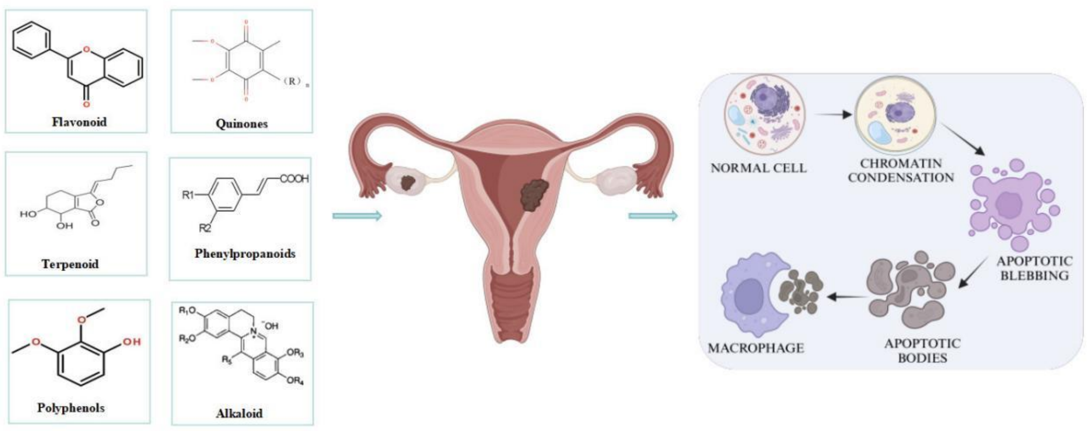

Supplementary Figure: Graphic Summary

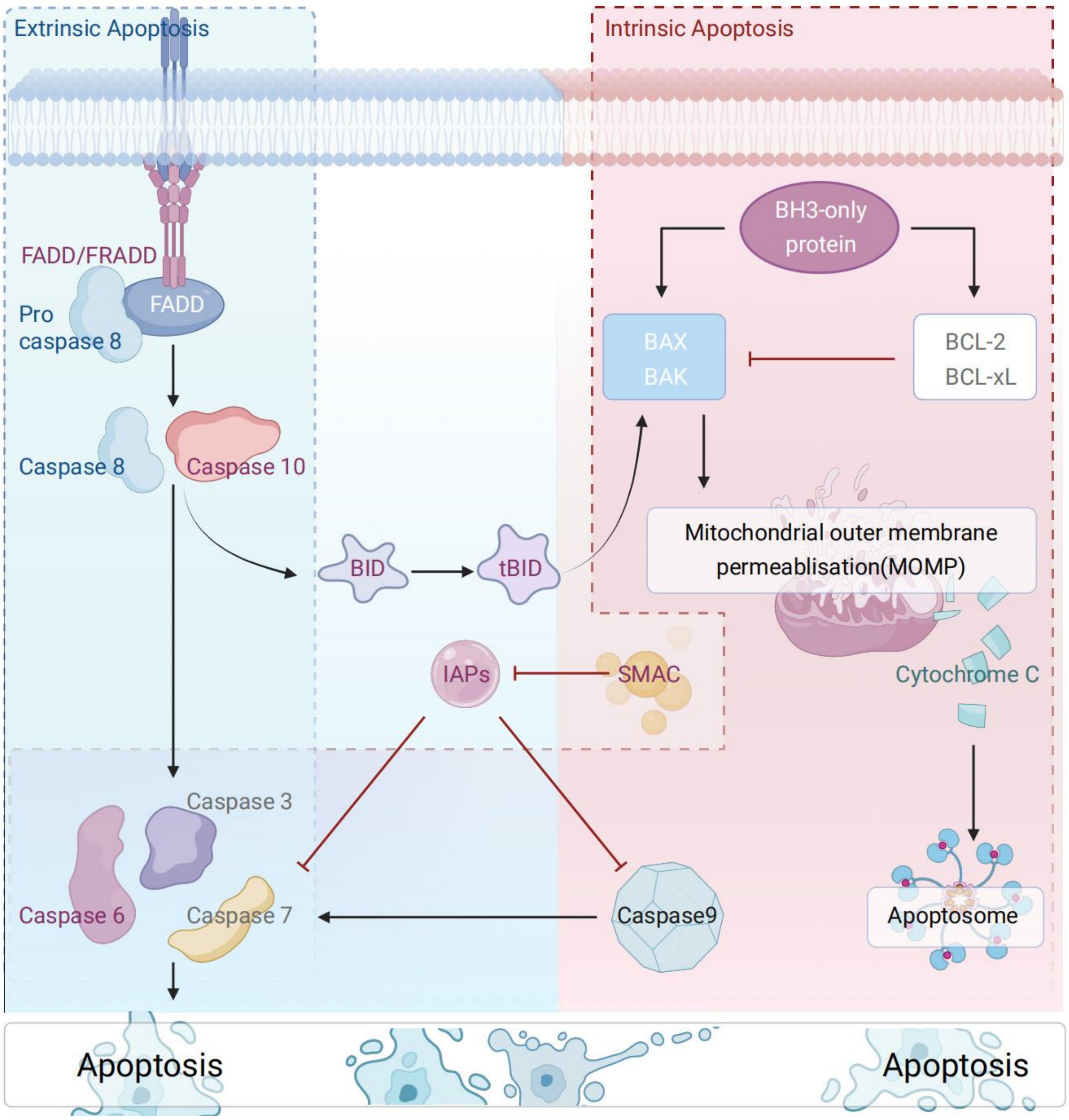

Supplementary Figure 1. Schematic diagram of the molecular mechanism of apoptosis.

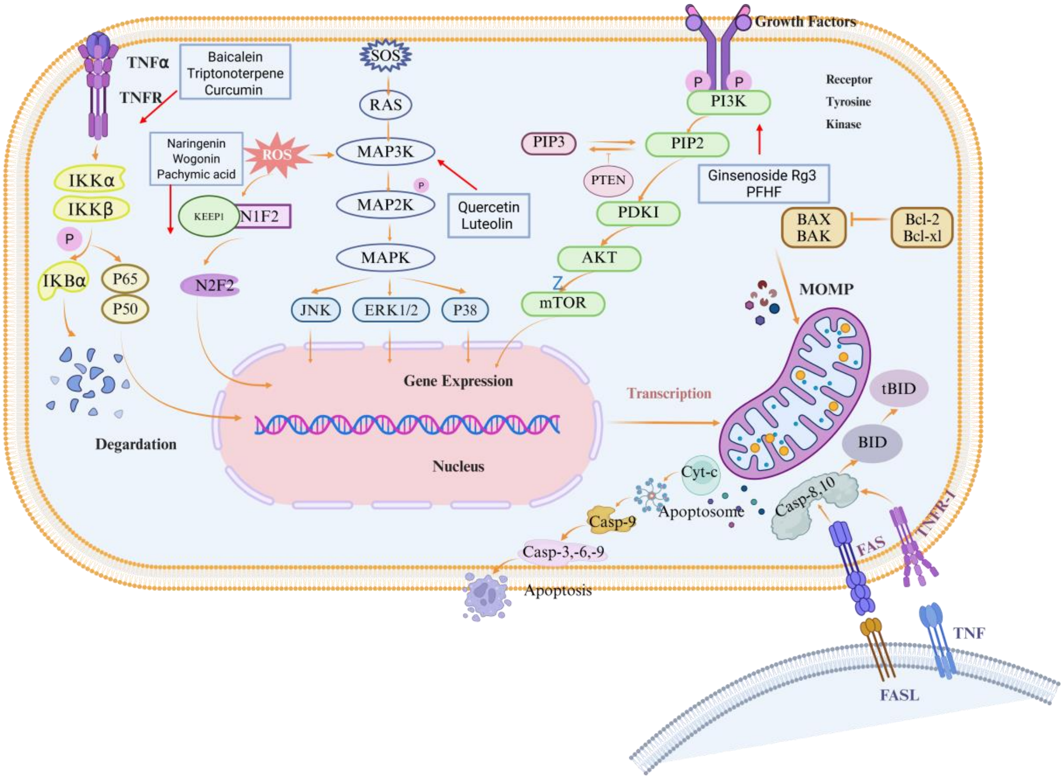

Supplementary Figure 2. Schematic diagram of molecular mechanisms of natural metabolites for the prevention and treatment of EMs by promoting apoptosis.

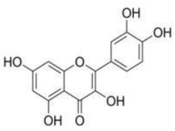
1.Quercetin

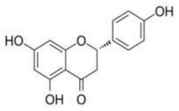
2.Naringenin

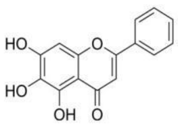
3.Baicalein

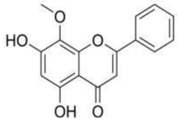
4.Wogonin

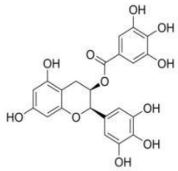
5.EGCG

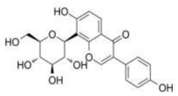
6.Puerarin

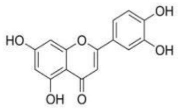
7.Luteolin

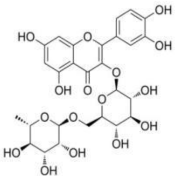
8.Rutin

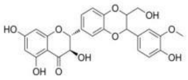
9.Silybin

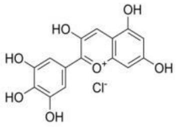
10.Delphinidin

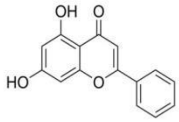
11.Chrysin

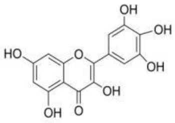
12.Myricetin

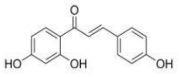
13.Isoliquiritigenin

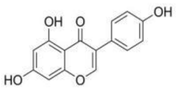
14.Genistein

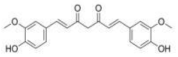
15.Curcumin

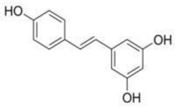
16.Resveratrol

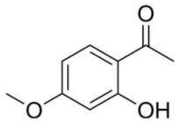
17.Paeonol

Supplementary Figure 3. The structural formula of polyphenols.

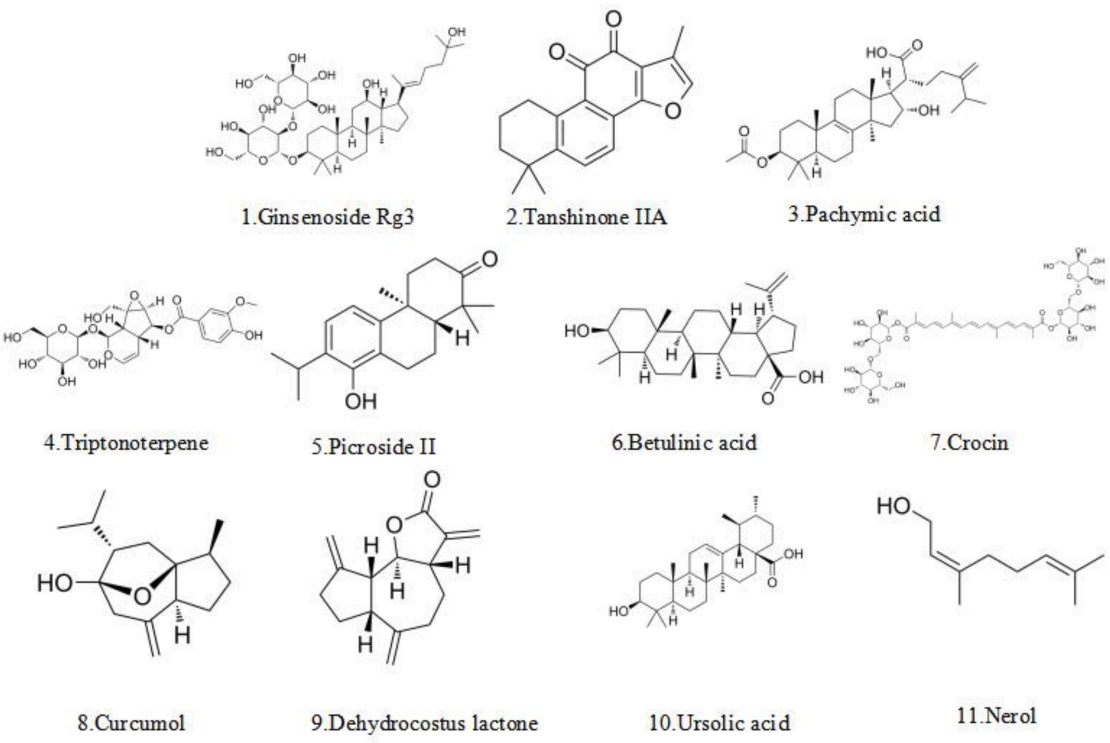

Supplementary Figure 4. The structural formula of terpenoid.

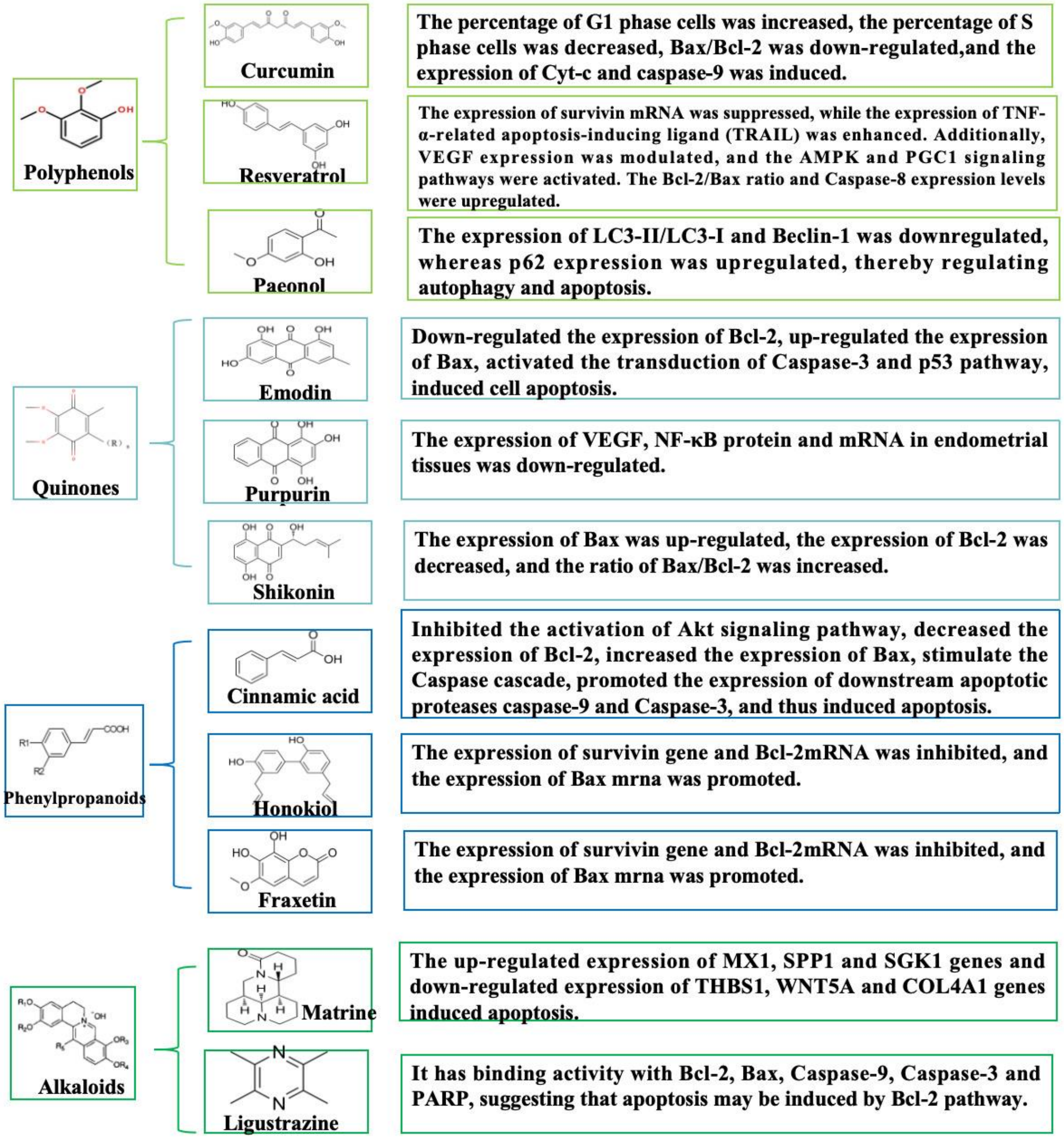

Supplementary Figure 5. Schematic diagram of the mechanism of quinones, phenylpropanoids and alkaloids in the treatment of EMs by promoting apoptosis.

1.2 Supplementary Tables

TABLE 1. Potential mechanism of polyphenols-induced apoptosis in the treatment of EMs.

Compound	In vitro /In vivo	Experimental model	Concentration	Pharmacological effects	Obstacles to development
Quercetin	In vitro /In vivo	VK2/E6E7 cells<br>End1/E6E7 cells<br>C57BL/6 mices	0,5,10,20 µM<br>35 mg/kg	Inhibition of ERK1/P38/MAPK/AKT signaling pathway induces G0/G1 cycle arrested and promoted apoptosis in cells	Low water solubility and chemical instability
	In vitro	Endometrial stromal cells	25 µM	Mediated AKT-ERK-p53 signaling pathway increased apoptosis and aged-like phenotype of endometrial stromal cells.	
	In vitro	End1/E6E7 cells	10,20,30,40 µg/mol	Upregulation of miR-340-5p expression level significantly reduces cell OD value and PCNA, Bcl-2 protein levels, increased cell apoptosis rate, and protein expression of Bax and Caspases3.	
	In vitro	End1/E6E7 cells	20µg/mol	Upregulation of Bax expression and inhibition of Bcl-2 expression in combination with CADM1 induced an increase in Sub G0/G1 phase cells and a decrease in G0/G1 phase cells.	
Naringenin	In vitro	VK2/E6E7 cells<br>End1/E6E7 cells	0, 5, 10, 20, 50,100 µM	Mitochondrial membrane depolarization and ROS production induced cell apoptosis	Low bioavailability and significant first-pass effect
	In vitro	SD rats	0.5µM, 1.0µM, 5.0µM	Inhibition of Nrf2/Keap1/HO1 signaling pathway induced mitochondrial membrane potential loss and ROS	

				production, leading to cell apoptosis	
Baicalein	In vitro	Endometrial stromal cells	0, 5, 10, 20, 40, 80, 160 μM	Activation of the NF-κB signaling pathway promoted cell cycle arrest in the G0/G1 phase and downregulated the expression of Bcl-2, PCNA, and cyclin D1 proteins.	Low lipophilicity and hydrophilicity, significant first-pass effect, low intestinal absorption
	In vitro /In vivo	Human ovarian endometriotic stromal cells	0-5 μg<br>40 mg/kg	Inhibited the MAPK/PI3K signaling pathway, increased mitochondrial calcium flux, induced mitochondrial depolarization and ROS, and promoted cell apoptosis	
Wogonin	In vitro /In vivo	Telomerase-immortalized Human Endometrial Stromal cells BALB/c mices	40,80,160μM<br>20 mg/kg	Induced cell cycle arrest in the G2/M phase, increased intracellular ROS accumulation, and inhibited the expression of estrogen receptor alpha in cells.	Low bioavailability and carrier stability check
	In vivo	SD rats	2mg/kg, 14mg/kg	Mediated the SIRT1/Nrf2 signaling pathway, up-regulated the protein expression levels of SIRT1, Nrf2, GPX4, FTL, and SLC7A11,inhibited ferroptosis, and induced apoptosis	
EGCG	In vivo	SCID mice	50 mg/kg	Inhibited the expression of VEGF-A, HIF-1α, NF-κB, and MAP2K1 mRNA, reduced angiogenesis, and promoted cell apoptosis	Very low water solubility and fat solubility, insufficient oral bioavailability, and significant first-pass effect
	In vivo	BALB/c mices	20 mg/kg	Inhibited cell proliferation, reduced angiogenesis, and induced apoptosis	
	In vivo	Immunocompromised mices	50 mg/kg	Inhibited the growth of ectopic lesions and the functional and structural microvessels within the lesions, promoted apoptosis of the lesions.	

	In vivo	C57BL/6 mices	50 mg/kg	Mediated Akt signaling pathway, inhibited angiogenesis, and induced apoptosis	
	In vito	SD rats	80 mg /kg	Reduced E2 levels in ectopic endometrial tissue, up-regulated ERβ expression, inhibited inflammatory processes, and promoted apoptosis.	
Puerarin	In vitro	Endometriotic stromal cells	1×10^{-6},5×10^{-6}, 1×10^{-5},5×10^{-5}, 1×10^{-4},5×10^{-4} mol/L	Promoted the recruitment of estrogen receptors and restricted the recruitment of coactivators in ESCs, thereby down-regulated the transcription of cyclin D1 and cdc25A, inhibited cell proliferation, and promoted cell apoptosis	Low solubility, low permeability, very low bioavailability, very low bioavailability
	In vitro	Endometrial stromal cells	100μmol/L	Up-regulated the gene expression of BAD, BAX, CASP8, CASP9, TNFRSF6, CDKN1B, CDKN2A, IFNA1, and IFNB1, downregulated the gene expression of FOS, CHEK2, SRC, ITGB5, MMP9, PDGFA, and NFKBIA, inhibited the formation of neovascularization in ectopic lesions, and promoted cell apoptosis	
	In vitro	Human endometriotic 12Z cells	0, 15, 30, 60 μM	Stimulated the activation of Caspase-8, Caspase-9, and Caspase-3 in endometriosis cells and hindered the selective activation of macrophages	
Luteolin					Low solubility, high permeability
	In vitro /In vivo	VK2/E6E7 cells End1/E6E7cells C57BL/6 mices	0, 5, 10, 20, 50, 100μM 40 mg/kg	Regulated the expression of PI3K/AKT and MAPK signaling proteins as well as CCNE1, blocked the cell cycle, inhibited cell proliferation,	

				increased DNA fragmentation, and induced cell apoptosis.	
Rutin	In vivo	Wistar albino rats	3000, 6000 µg/kg	Downregulation of Bcl-2, upregulation of Bax and caspase9 expression induced cell apoptosis, and improved oxidative stress by reduced MDA concentration and increased SOD, GPx, and TAC concentrations	Low intestinal absorption and poor oral bioavailability
	In vitro	CRL-7566 cells	70 µM	Targeted NOX4 and inhibited ROS/HIF-1 α signaling pathway, affected the malignant biological behavior of cells.	
Silybin	In vitro /In vivo	VK2/E6E7 cells End1/E6E7cells C57BL/6 mice	0, 2.5,5,10,25,50 µM 100 mg/kg	Promoted cell cycle arrest, oxidative stress, lipid peroxidation, and endoplasmic reticulum stress, thereby inducing cell apoptosis	Poor oral absorption
Chrysin	in vitro	VK2/E6E7 cells End1/E6E7 cells	0, 5, 10, 20, 50,100 µM	Stimulated endoplasmic reticulum stress, ROS production, and cytoplasmic calcium levels, downregulated PI3K signaling pathway transduction, and induced cell apoptosis	Low water solubility and first pass effect
Myricetin	In vitro /In vivo	VK2/E6E7 cells End1/E6E7 cells<br><br>C57BL/6 mices	0, 5, 10, 20, 50,100 µM<br><br>29 mg/kg	Downregulated the phosphorylation of ERK1/2 and PI3K/AKT signaling pathways, promoted G0/G1 phase arrest of cell cycle, inhibited cell proliferation, promoted mitochondrial dysfunction, accumulation of reactive oxygen species and calcium ions, and induced cell apoptosis.	Low solubility and high rate of intestinal metabolism
Delphinidin	In vitro	VK2/E6E7 cells End1/E6E7 cells	0, 20, 50,100 µM	Affected mitochondrial membrane potential and increased cytoplasmic calcium	Restricted intestinal absorption

				levels, thereby inducing cell apoptosis	
Isoliquiritigenin	In vitro /In vivo	End1/E6E7 cells Balb/c mices	0, 25, 50, 75,100 µM 1mg/kg,5 mg/kg	Inhibited the expression of Bcl-2 and increased the expression of Bax in endometriosis lesions, activated Caspase-3, promoted cell apoptosis, and inhibited the growth of endometriosis lesions	Poor targeting
Genistein	In vivo	SD rats	50,150,450mg/kg/d	It may be related to the down-regulation of Bcl-2, up-regulation of Bax, and other related apoptotic factors, and at the same time, it inhibited other malignant activities, such as invasion and vascular proliferation of ectopic endometrium by inhibiting the expression of VEGF, CD34, COX-2, and MMP-9/TIMP-1.	Low oral absorption
Curcumin	In vitro	Human ectopic endometrial cells	50 μmol/l	The percentage of cells in G1 phase increased and the percentage of cells in S phase decreased.	Low water solubility, poor gastrointestinal stability, and rapid metabolism
	In vivo	Balb/c mices	12, 24, 48 mg/kg	Decreased Bax/Bcl-2 and induced the expression of Cyt-C and Caspase-9.	
	In vivo	Balb/c mices	50mg/ml	Downregulated of VEGF expression.	
Resveratrol	In vitro	Human ectopic endometrial stromal cells	40–120µM	Inhibits survivin mRNA expression and enhances trail, an apoptosis inducing ligand associated with TNF-α.	Poor water solubility, poor photostability and strong first pass effect.
	In vitro	Human ectopic endometrial stromal cells	100 µM	Increased Bcl-2/Bax gene expression.	
	In vivo	SD rats	15 , 45 mg/kg/d	Increased the expression level of Caspase-8, activated PPARα, and upregulated	

				AMPK signaling and pcg1 pathway.	
Paeonol	In vitro	Human ectopic endometrial stromal cells	0、10、30、50、100μM	Downregulated LC3-II/LC3-I and Beclin-1, while upregulated p62.	Poor water solubility, short peak time and fast drug absorption.

TABLE 2. Potential mechanism of terpenoids induced apoptosis in the treatment of EMs

Compound	In vitro /In vivo	Experimental model	Concentration	Pharmacological effects	Obstacles to development
Ginsenoside Rg3	In vitro	Ectopic endometriotic stromal cells	25,50,100,150 μg/ml	The mRNA expression of NF-κB P65,IL-8 and CIAP-2 was decreased to induce apoptosis	Poor water solubility, low bioavailability, easily degraded and difficult to optimize dosage
	In vitro	Endometrial cells	0.313×10^{4},0.625×10^{4},1.25×10^{4},2.5×10-4,3.75×10^{-4}mol/L	Induced cell G0/G1 cycle arrest and promoted cell apoptosis	
	In vitro	Ectopic endometriotic stromal cells	0,25,50,100,150 μg/mL	Up-regulated the expression of Caspases3 and down-regulated the expression of VEGF	
	In vivo	SD rats	5mg/kg,10mg/kg	The PI3K/Akt/mTOR signaling pathway was blocked, and the expression of VEGF, p-Akt and p-mTOR was down-regulated	
Tanshinone IIA	In vivo	SD rats	30 mg/kg	TGF-β/SMADS signaling pathway was inhibited, Vegf, Mmp9 and Bcl2 mRNA expressions were down-regulated, and Bax and Caspase9 mRNA expressions were up-regulated	Poor water solubility, low bioavailability and difficulty in dose optimization
	In vivo	SD rats	10,20,30mg/kg	It inhibited the expression of Bcl-2 protein and promoted the	

				expression of Bax and Caspase-9 protein	
	In vitro	Ectopic endometrial stromal cells	10,20,40,80,160μM	Apoptosis was mediated by a dependent reduction of 14-3-3 ζ	
Sodium tanshinone IIA sulfonate	In vivo	Balb/C mice	40–80 mg/day	The activation of p53, Sav1, and CCN1, coupled with the inhibition of HAS2, GM-CSF, and other cytokines, promoted cellular apoptosis	
Pachymic acid	In vivo	Sprague-Dawley rats	3.5 mg/kg,7.0 mg/kg	Activated AMPK/GSK-3β/Nrf2 signaling pathway, reduced MDA, TNF-α and IL-6 levels, inhibited the expression of ACSL4 and PTGS2 proteins, and promoted the expression of GSH, p-AMPK/AMPK, p-GSK-3β/GSK-3β and Nrf2	Low levels in nature, low bioavailability, and toxicity and dose relationship not yet defined
Picroside II	In vivo	SD rats	5mg/kg,10mg/kg,30 mg/kg	The expression of Bax, Bcl-2, VEGF and TGF-β was inhibited, and the number of CD206+ macrophages was decreased	Low bioavailability, toxicity and dose relationships have not been clarified
Triptonoterpene	In vitro	Endometrial stromal cells	20,40 μg/mL	Increased KISS-1 mRNA expression, inhibited Ki67 and Pro-Caspase3 expression, promoted Cleaved Caspase3 and Bax protein expression	Poor water solubility, low bioavailability, and difficult dose optimization
Betulinic acid	In vitro	Human Endometriotic Epithelial Cell Line 12Z, Primary endometriotic epithelial cells	0,5,10,15,20,25,30,35,40μM	Targeted genes associated with Estrogen Receptor β, including SOD2, NRF, COX2, and MMP1, effectively inhibited the production of pro-inflammatory cytokines	Low solubility, low bioavailability, and difficult dose optimization
Neroli oil	Bitter orange tree	In vivo	40mg/kg	Increased SOD, CAT levels, decreased NO, TNF-α, IL-8, IL-10,VEGF levels	Low water solubility, first pass metabolism, and

					potential toxicity of metabolites
Crocin	In vitro/<br>In vivo	Human monocyte THP-1 cell<br>Balb/c mice	20 μM<br>25 mg/kg	Reduced the release of VFP, IL-6, TNF-A and other cytokines, and induced cell apoptosis	Insufficient stability, low bioavailability
Curcumol	In vitro/<br>In vivo	Ectopic endometrial stromal cells<br>Sprague-Dawley rats	5–40 μg/L<br>20 mg/kg	Targeted JAK2/STAT3 pathway, inhibited the expression of Bax, caspase-3, TNF-α, IL6 and ILL-1L, and promoted the expression of Bcl2 protein	Low solubility, low bioavailability, and difficult dose optimization
Dehydrocostus lactone	In vitro	Human endometriotic cell line (12Z)	5, 10, 20 μM	Targeted the Akt and NF-κB pathways leads to the activation of caspase-3, caspase-8, and caspase-9, a reduction in the production of BDNF, NGF, NT-3, and NT-4/5, and an inhibition of the expression of macrophage M2 markers, including IL-10, VEGF, MMP-2, and MMP-9	Low water solubility, low bioavailability, non-selective binding, and difficult dose optimization
Ursolic acid	In vitro	Ectopic endometrial stromal cells	15,30,45,60μM	Promoted the activity of caspase-3 and inhibited the expression of COX-2, PGE2 and VEGF	Poorly soluble and rapidly metabolized in the liver and gastrointestinal tract

TABLE 3. Clinical trials of traditional natural metabolites for treating EMs registered on ClinicalTrials.gov

Study title	NCT number	Status	Phase	Research progress
Evaluating the Impact of a Novel Cannabinoid Product for Endometriosis	NCT06477406	Recruiting	Phase II	The pain score (VAS), Beck Anxiety Inventory (BAI) and Beck Depression Inventory (BDI) of patients with cannabinoid endometriosis decreased significantly after use. The

				inflammatory factors IL-1, IL-6, IL-8, TNF-α were significantly decreased.
Effect of Quercetin Supplementation on Endometriosis Outcomes	NCT05983224	Recruiting	Not Applicable	---
Cannabidiol for the Treatment of Pelvic Pain in Endometriosis (DREAMLAND)	NCT05670353	Recruiting	Phase III	The intensity of pain was significantly reduced, the change of pain threshold was significantly improved, and the scores of generalized anxiety disorder (GAD7) scale and depressive symptoms were decreased; There was no significant change in the concentration of alanine aminotransferase (ALT), aspartate aminotransferase (AST) and bilirubin in plasma.
Cannabidiol and Management of Endometriosis Pain	NCT04527003	Terminated	Phase III	The pain intensity measured by cannabidiol visual analog scale was significantly improved.
Green Tea Extract for Endometriosis Treatment	NCT02832271	Completed	Phase II	Green tea extract can reduce endometriosis lesions, reduce oral Rating Scale (ESS) and visual analogue scale (VAS) scores, and reduce the total number of new blood vessels in pathological tissues, without obvious side effects.
Pertubation With Lignocaine in Endometriosis	NCT01329796	Completed	Phase II	Lignocaine can significantly reduce the visual analogue scale (VAS), however, the quality of life questionnaire remains to be evaluated.

Note: As of February 16, 2025, a search for "condition/disease" on https://clinicaltrials.gov/ using the keyword "Endometriosis" returned 756 studies. Among these, 6 clinical trials pertaining on natural products were identified in the context of EMs treatment.
